# TMEM41B and VMP1 modulate cellular lipid and energy metabolism for facilitating dengue virus infection

**DOI:** 10.1371/journal.ppat.1010763

**Published:** 2022-08-08

**Authors:** Meisam Yousefi, Wai Suet Lee, Biaoguo Yan, Liang Cui, Cythia Lingli Yong, Xin Yap, Kwan Sing Leona Tay, Wenjie Qiao, Dewei Tan, Nur Insyirah Nurazmi, Martin Linster, Gavin J. D. Smith, Yie Hou Lee, Jan E. Carette, Eng Eong Ooi, Kuan Rong Chan, Yaw Shin Ooi

**Affiliations:** 1 Emerging Infectious Diseases Program, Duke-NUS Medical School, Singapore, Singapore; 2 Antimicrobial Resistance Interdisciplinary Research Group, Singapore-MIT Alliance for Research and Technology, Singapore, Singapore; 3 Department of Microbiology and Immunology, Stanford University School of Medicine, Stanford, California, United States of America; 4 KK Research Centre, KK Women’s and Children’s Hospital, Singapore, Singapore; 5 Saw Swee Hock School of Public Health, National University of Singapore, Singapore, Singapore; University of North Carolina at Chapel Hill School of Medicine, UNITED STATES

## Abstract

Transmembrane Protein 41B (TMEM41B) and Vacuole Membrane Protein 1 (VMP1) are two ER-associated lipid scramblases that play a role in autophagosome formation and cellular lipid metabolism. TMEM41B is also a recently validated host factor required by flaviviruses and coronaviruses. However, the exact underlying mechanism of TMEM41B in promoting viral infections remains an open question. Here, we validated that both TMEM41B and VMP1 are essential host dependency factors for all four serotypes of dengue virus (DENV) and human coronavirus OC43 (HCoV-OC43), but not chikungunya virus (CHIKV). While HCoV-OC43 failed to replicate entirely in both TMEM41B- and VMP1-deficient cells, we detected diminished levels of DENV infections in these cell lines, which were accompanied by upregulation of the innate immune dsRNA sensors, RIG-I and MDA5. Nonetheless, this upregulation did not correspondingly induce the downstream effector TBK1 activation and Interferon-beta expression. Despite low levels of DENV replication, classical DENV replication organelles were undetectable in the infected TMEM41B-deficient cells, suggesting that the upregulation of the dsRNA sensors is likely a consequence of aberrant viral replication rather than a causal factor for reduced DENV infection. Intriguingly, we uncovered that the inhibitory effect of TMEM41B deficiency on DENV replication, but not HCoV-OC43, can be partially reversed using exogenous fatty acid supplements. In contrast, VMP1 deficiency cannot be rescued using the metabolite treatment. In line with the observed phenotypes, we found that both TMEM41B- and VMP1-deficient cells harbor higher levels of compromised mitochondria, especially in VMP1 deficiency which results in severe dysregulations of mitochondrial beta-oxidation. Using a metabolomic profiling approach, we revealed distinctive global dysregulations of the cellular metabolome, particularly lipidome, in TMEM41B- and VMP1-deficient cells. Our findings highlight a central role for TMEM41B and VMP1 in modulating multiple cellular pathways, including lipid mobilization, mitochondrial beta-oxidation, and global metabolic regulations, to facilitate the replication of flaviviruses and coronaviruses.

## Author summary

Given the concerns over potential global health burdens imposed by endless emerging and re-emerging viruses as well as the limited therapeutic options to intervene, host-directed therapeutics can serve as a promising approach to broadly prepare for future pandemics. TMEM41B and VMP1 have been demonstrated as essential host factors for at least two unrelated groups of clinically important RNA viruses with outbreak potential. Therefore these ER membrane proteins could potentially serve as cellular targets for developing host-directed therapeutics. However, the effort must be first supported by a comprehensive understanding of their function in viral infection. Here, we dissected the role of TMEM41B and VMP1 in dengue virus infection, showing that both these proteins are crucial for the normal functionality of mitochondria and the regulation of cellular metabolites. We further provided evidence that these metabolic roles contribute to TMEM41B and VMP1 essentiality in dengue virus infection.

## Introduction

DENV is a clinically important flavivirus endemic to the tropical and sub-tropical world. It is the most prevalent mosquito-borne viral infection in humans, with estimations of 300–400 million infected individuals annually [1]. There are no specific antiviral therapeutics against DENV, and the only licensed vaccine is limited to individuals with pre-existing DENV immunity [2,3]. Deciphering the host factors that promote or restrict DENV infection and replication is essential for developing effective therapeutics against the virus [4]. Hitherto, several independent studies including multiple genetic screenings have shown that the endoplasmic reticulum (ER) and a subset of ER-associated proteins, e.g., the ER translocon, the ER membrane protein complex (EMC), and the oligosaccharyltransferase (OST) complex, have critical roles in DENV infection and pathogenesis [5–8]. Some of these ER-associated proteins can be broadly exploited by multiple viruses [9,10], such as Transmembrane Protein 41B (TMEM41B) and Vacuole Membrane Protein 1 (VMP1) [5,7,11–14].

TMEM41B was previously identified as a novel autophagy factor that modulates autophagosome formation [15–17]. Interestingly, a functional association between TMEM41B and VMP1 has been hinted by previous reports showing that TMEM41B deficiency can be partially rescued by VMP1 overexpression [15,17]. These two ER membrane proteins also share a structural homology by harboring a functional VTT transmembrane domain [12,17]. In addition, both TMEM41B- and VMP1-deficient cells displayed enlarged lipid droplets, an observation which has been provisionally attributed to lipid accumulation due to a disrupted release of free fatty acids (FAs) from lipid droplets to other organelles such as mitochondria [15,16]. More recently, both TMEM41B and VMP1 were characterized as ER scramblases that can shuttle phospholipids between bilayer membrane leaflets [18–21].

Most virus infections are typically accompanied by broad metabolic alterations in the host cells. Host cell metabolic pathways, such as autophagy, beta-oxidation, and glycolysis, are often hijacked to promote productive virus replication and evade host immune responses [22]. For example, DENV infection can reshape glycolytic activity and lipid metabolism [23–26]. Heaton *et al*. have previously demonstrated that DENV can induce lipophagy in the host cells to mobilize FAs from lipid droplets to fuel mitochondrial beta-oxidation and ATP production. Intriguingly, they have shown that reduced DENV replication due to genetic deficiencies in autophagy factors can be metabolically rescued by exogenous FA complementation [27]. Since TMEM41B and VMP1 are implicated in autophagy and lipid metabolism, it would be crucial to determine whether there are any links between the cellular functions of these ER membrane proteins and DENV infection. Interestingly, TMEM41B and VMP1 were recently identified as coronavirus host factors that play a role in the formation of replication organelles [11,13,14,28]. However, whether or not TMEM41B plays a universal role in flavivirus and coronavirus infections remains to be elucidated.

Here, we dissected the molecular mechanisms behind the essentiality of TMEM41B and VMP1 in the context of DENV infection, with cross-comparisons to CHIKV and HCoV-OC43. We investigated the role of TMEM41B and VMP1 in viral replication by assessing virus-induced innate immune RNA sensing and FA complementation. We next examined the effects of TMEM41B and VMP1 deficiencies on mitochondrial beta-oxidation and performed mass spectrometry-based profiling of cellular lipids and metabolites to comprehensively understand the global changes in the metabolome of TMEM41B- and VMP1-deficient cells. Our study strongly indicates distinctive roles for TMEM41B and VMP1 in regulating cellular metabolism to facilitate DENV infection.

## Results

### TMEM41B and VMP1 are host dependency factors for all DENV serotypes and HCoV-OC43, but not CHIKV

**Fig 1:**
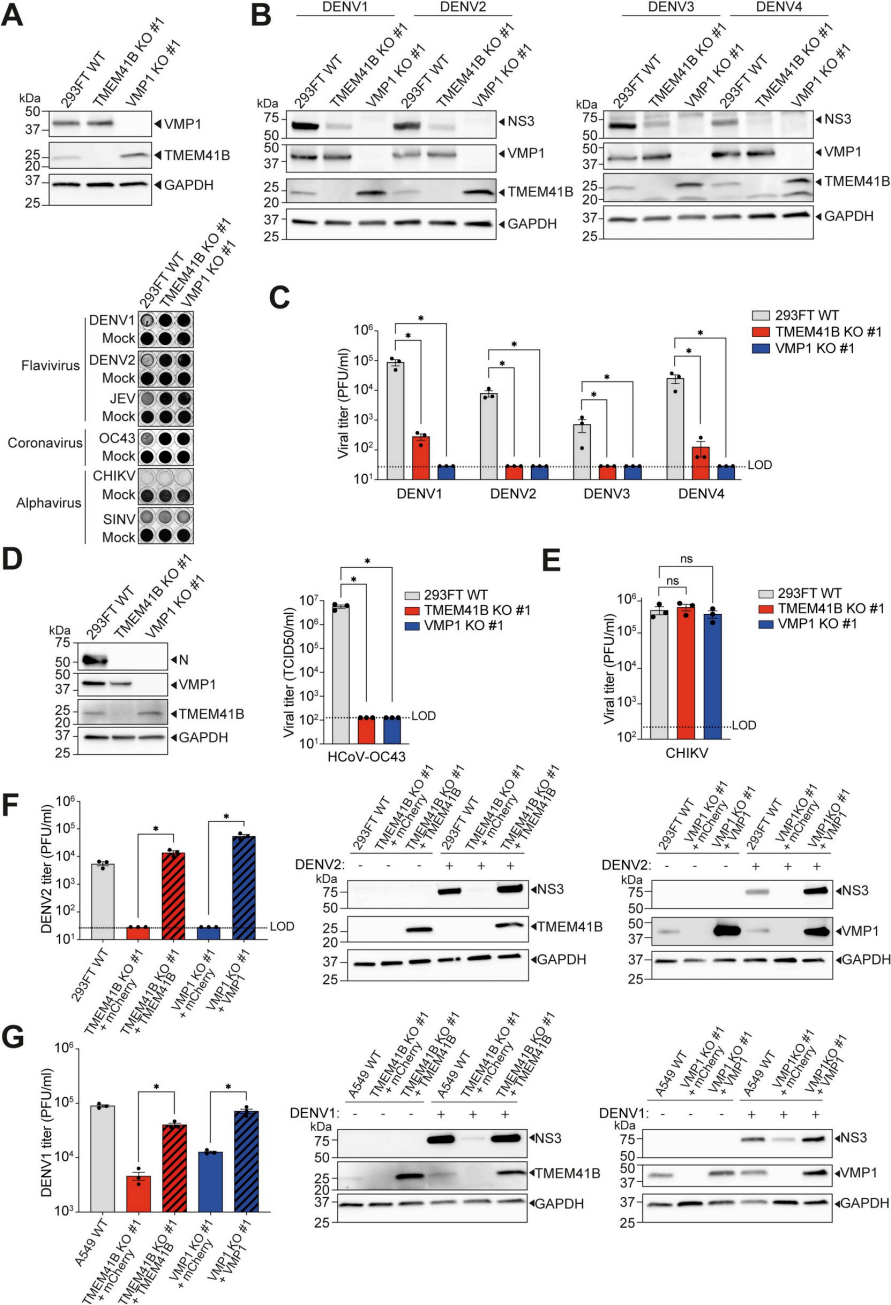
TMEM41B and VMP1 are host dependency factors for flaviviruses and human coronaviruses, but not alphaviruses. **(A)** Multiple rounds of virus infection in 293FT WT, TMEM41B KO clone #1, and VMP1 KO clone #1 cells. (Top) Western blotting analysis to detect TMEM41B and VMP1 protein levels in the WT and KO cells. (Bottom) Crystal violet staining to visualize surviving cells after cytolytic virus infection. Non-infected controls are indicated as “mock”. **(B, C)** Validation of TMEM41B and VMP1 as host dependency factors across all DENV serotypes. For (B), cells were infected by either DENV1 (MOI = 1 PFU/cell), DENV2 (MOI = 0.1 PFU/cell), DENV3 (MOI = 3 PFU/cell), or DENV4 (MOI = 5 PFU/cell) for 72 hours. Accumulation of DENV NS3 proteins was detected by western blot. For (C), cells were infected at MOI of 1 for 72 hours. Released progeny virions in the supernatant were assessed by plaque assay. **(D)** HCoV-OC43 protein accumulation (Left) and progeny virion levels (Right) in 293FT WT and KO cells. Cells were infected with HCoV-OC43 at MOI of 0.5 for 48 hours. Released progeny virions in the supernatants were harvested for titration by TCID_50_ assay, and infected cell lysates were analyzed by western blotting. **(E)** Production of CHIKV progeny virions from WT and KO cells. Cells were infected with CHIKV at MOI of 0.1 for 24 hours, and released virions were harvested for titration by plaque assay. **(F)** cDNA complementation rescue of DENV infection. (Left) Production of DENV2 infectious particles from KO cells complemented with corresponding cDNAs, i.e., mCherry (control), *TMEM41B*, or *VMP1*. (Right) Western blotting analysis of DENV2 NS3 accumulation in WT, KO, and cDNA-complemented cells. Cells were infected at MOI of 0.1 for 72 hours. Released virions in the supernatants were harvested for titration by plaque assay, and cell lysates were analyzed by western blotting. **(G)** Validation of TMEM41B and VMP1 as DENV host factors in A549 cells. (Left) Production of DENV1 infectious particles from A549 WT, TMEM41B KO clone #1, and VMP1 KO clone #1 cells complemented with corresponding cDNAs. (Right) Western blotting analysis of DENV1 NS3 accumulation in WT, KO, and cDNA-complemented cells. Cells were infected at MOI of 0.5 for 32 hours. GAPDH was used as a loading control for all blots. All data shown represent results from at least two independent experiments. Error bars represent mean +/- SEM, n = 3. * indicates *p-value* < 0.05 as determined by two-tailed unpaired t-test or one-way ANOVA. LOD indicates the limit of detection.

To validate the pro-viral role of TMEM41B and VMP1 that we initially observed in CRISPR screens for all DENV serotypes (DENV1-4) [5,7], we generated single cell-derived clonal (hereby referred to as “clonal”) TMEM41B- and VMP1-deficient human embryonic kidney 293FT cells using CRISPR-Cas9 genome editing (Fig 1A and S1 Table). We first challenged these clonal knockout (KO) cell lines with cytolytic infections of flaviviruses, i.e., DENV1, DENV2, and Japanese encephalitis virus (JEV). To cross-compare the phenotypes, these cell lines were challenged in parallel with two other groups of enveloped RNA viruses–HCoV-OC43 and alphaviruses, CHIKV and Sindbis virus (SINV). Compared to the wild-type (WT) cells, we observed that both TMEM41B KO and VMP1 KO cells were wholly protected from cytolytic infection of flaviviruses and HCoV-OC43, but not alphaviruses (Fig 1A). This was consistent with recent reports showing DENV2 and HCoV-OC43 infect significantly fewer TMEM41B- and VMP1-deficient human cells as compared to WT [11,12]. We then determined the magnitude of viral replication reduction in TMEM41B KO and VMP1 KO cells by visualizing the accumulation of viral proteins in infected cells and measuring the production of infectious progeny virions. Interestingly, regardless of DENV serotypes, we observed a drastic decrease in viral protein accumulation (represented by non-structural protein 3, NS3) in TMEM41B KO and VMP1 KO cells (Fig 1B). Correspondingly, we detected ~2–3 logs decrease in the release of infectious DENV virions from these KO cells (Fig 1C). A more pronounced impact was observed for HCoV-OC43 infection, as we failed to detect any viral protein accumulation (represented by nucleocapsid protein, N) and production of infectious progeny virions in the absence of TMEM41B and VMP1 (Fig 1D). Consistent with the results presented in Fig 1A, we could not detect any differences between CHIKV infected WT and KO cells (Fig 1E), corroborating that TMEM41B and VMP1 are dispensable for alphavirus infection. To rule out potential off-target effects that might be caused by CRISPR-Cas9 genome editing, we complemented the KO cell lines using *TMEM41B* cDNA and *VMP1* cDNA. Upon the cDNA complementation, DENV infection was rescued to levels comparable to the WT cells, reinforcing that both TMEM41B and VMP1 deficiencies are indeed responsible for the observed diminished levels of viral infections (Fig 1F). Similar results were observed in other clonal TMEM41B KO and VMP1 KO cells (S1A Fig), indicating that these phenotypes were not influenced by any clonality issues. We further generated clonal TMEM41B KO and VMP1 KO cell lines using human lung carcinoma epithelial cells, A549 cells [29–31], to demonstrate that these phenotypes were not limited to only a specific cell type. As predicted, we observed a similar reduction in DENV protein accumulation and infectious progeny virion production in the TMEM41B KO and VMP1 KO cells. Again, the knockout phenotype was on-target as it could be reversed upon cDNA complementation (Fig 1G). In addition, no significant differences in cell doubling times were observed for the KO, and cDNA complemented cells in comparison with the WT cells, dismissing the possibility that diminished viral infection in the KO cells was due to defective cell growth (S1B Fig).

### Impaired DENV infection in TMEM41B- and VMP1-deficient 293FT and A549 cells induces upregulation of cellular dsRNA sensors

**Fig 2:**
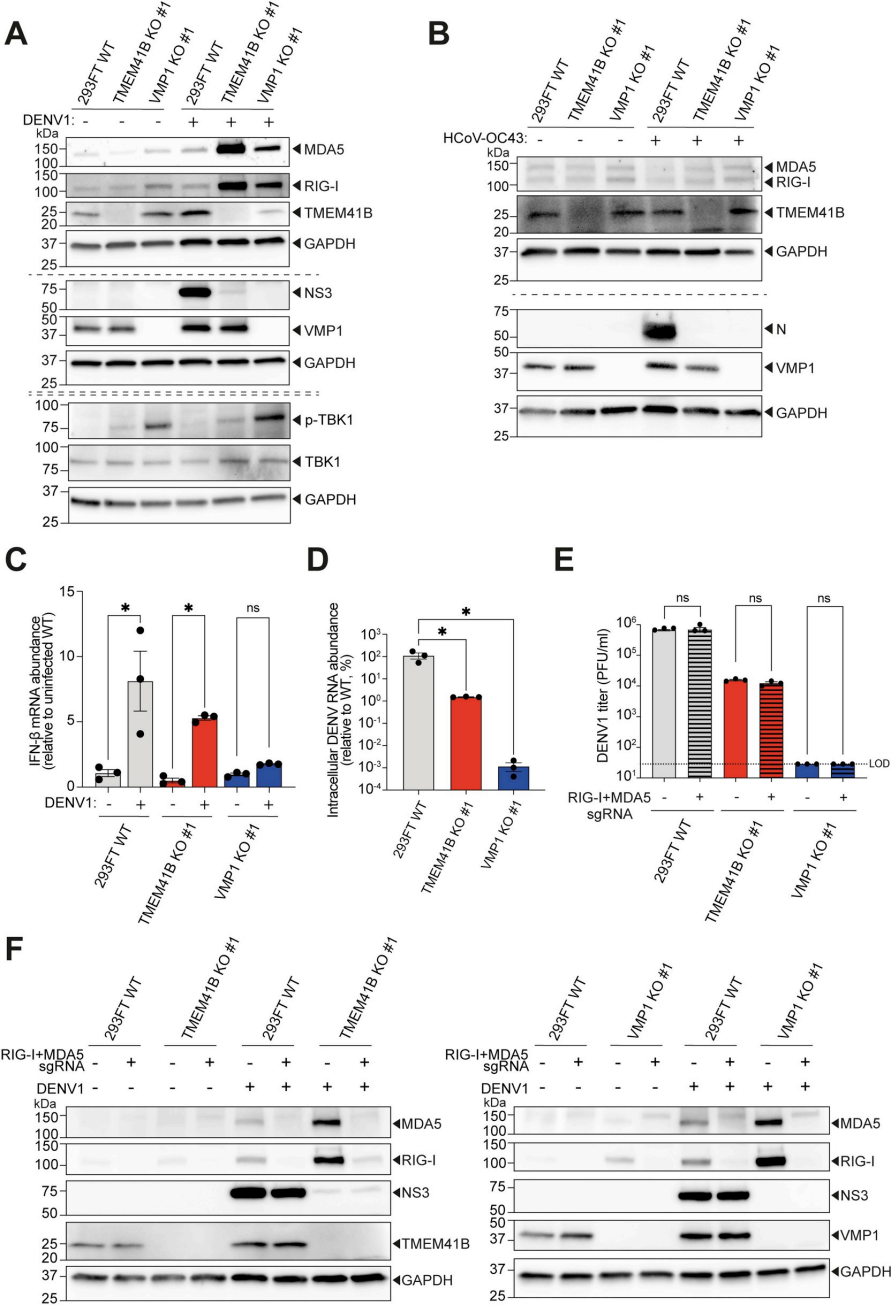
Upregulation of dsRNA sensors in 293FT TMEM41B KO and VMP1 KO cells upon DENV infection. **(A)** Western blotting analysis to detect RIG-I, MDA5, TBK1, and p-TBK1 protein levels in 293FT WT cells, TMEM41B KO clone #1, and VMP1 KO clone #1, upon DENV infection. Cells were infected with DENV1 at MOI of 1 and lysates were harvested at 48 hours post-infection. **(B)** Western blotting analysis to detect RIG-I and MDA5 protein levels in 293FT WT, TMEM41B KO clone #1, and VMP1 KO clone #1 cells, upon HCoV-OC43 infection. Cells were infected with HCoV-OC43 at MOI of 0.5 and lysates were harvested at 48 hours post-infection. **(C,D)** IFN-β mRNA (C) and DENV RNA (D) abundance in uninfected and DENV1 infected (MOI of 1, 48 hours post-infection) WT and KO cells. Values demonstrated are relative to the expression levels in uninfected WT cells (C), or DENV infected WT cells (D). **(E,F)** DENV viral protein accumulation and infectious viral particles produced in 293FT WT, TMEM41B KO clone #1, and VMP1 KO clone #1 cells, upon further knocking out of RIG-I and MDA5. Infection assays were carried out at MOI of 1 and (E) the amount of infectious virus in the supernatant was determined at 48 hours post-infection by plaque assay, and (F) protein levels of the indicated proteins were determined by western blotting analysis. GAPDH was used as a loading control for all blots. 18S rRNA was used as the control for the RT-qPCR experiments. All data shown represent results from at least two independent experiments. Single-dashed lines divide blots from two gels loaded with the same set of lysates. Double-dashed lines divide blots from gels belonging to separate experiments using the same experimental conditions. Error bars represent mean +/- SEM, n = 3. * indicates *p-value* < 0.05 as determined by one-way ANOVA. LOD indicates the limit of detection.

Flaviviruses and human coronaviruses are known to rewire the ER to form viral replication organelles, which facilitate viral RNA synthesis and assist the virus in evading cellular innate immune sensing, especially double-stranded RNA (dsRNA) sensors like RIG-I and MDA5 [32,33]. Hoffmann *et al*. have recently proposed TMEM41B as a key factor that facilitates flavivirus replication complex formation [12]. We wondered if the impaired viral replication observed in TMEM41B- and VMP1-deficient cells was due to the aberrant viral RNA replication that may trigger RIG-I and MDA5 activation [34]. While TMEM41B and VMP1 deficiencies did not lead to an increased protein expression of RIG-I and MDA5 endogenously, we observed a substantial upregulation of RIG-I and MDA5 in TMEM41B KO and VMP1 KO cells upon DENV infection as compared to WT (Figs 2A and S2). This observation suggested that the aberrant viral RNA replication was detected by these dsRNA sensors. Intriguingly, HCoV-OC43 did not induce activation of RIG-I and MDA5 in the KO cells, which could be due to more deleterious effects of TMEM41B and VMP1 deficiencies as suggested by a complete absence of viral proteins and progeny virions (Figs 1D and 2B). All these observations hinted that upregulation of RIG-I and MDA5 in TMEM41B- and VMP1-deficient cells might be concomitant with aberrant DENV RNA replication. To examine the impacts of RIG-I and MDA5 upregulation in the KO cells upon DENV infection, we assessed the activation of TBK1, a key downstream effector. TBK1 is a key mediator for multiple cellular pathways including innate immune signaling, and its activation is indicated by its phosphorylation (p-TBK1) [34,35]. Interestingly, we observed that TBK1 was activated in both TMEM41B KO and VMP1 KO cells prior to DENV infection (Fig 2A), while uninfected WT cells harbored undetectable level of p-TBK1. TBK1 activation was mildly elevated in the KO cells upon DENV1 infection. We further measured interferon-beta (IFN-β) expression levels, a reference for the induction of interferon responses upon DENV infection [36]. Although we observed an endogenous activation of TBK1 in the uninfected KO cells, upregulation of IFN-β expression was only triggered upon DENV infection (Fig 2C). Notably, the IFN-β expression levels correlated with the levels of DENV RNA loads (Fig 2D), rather than RIG-I, MDA5, or p-TBK1 (Fig 2A). These observations demonstrated that the upregulations of RIG-I and MDA5 did not directly elevate the canonical interferon responses, raising the question whether the upregulation of these dsRNA sensors is contributing to the reduced DENV replication in these KO cells. To address this question, we further deleted both *RIG-I* and *MDA5* in the 293FT WT, TMEM41B KO, and VMP1 KO cells. Surprisingly, MDA5 and RIG-I deficiencies (RIG-I and MDA5 double-KO) did not promote DENV replication in any of the conditions, indicating that the upregulation of the dsRNA sensors is unlikely to be the main contributing factor to the diminished viral infection in TMEM41B KO and VMP1 KO cells (Fig 2E and 2F).

### DENV infection in TMEM41B-deficient 293FT cells can be partially rescued using exogenous fatty acids

**Fig 3:**
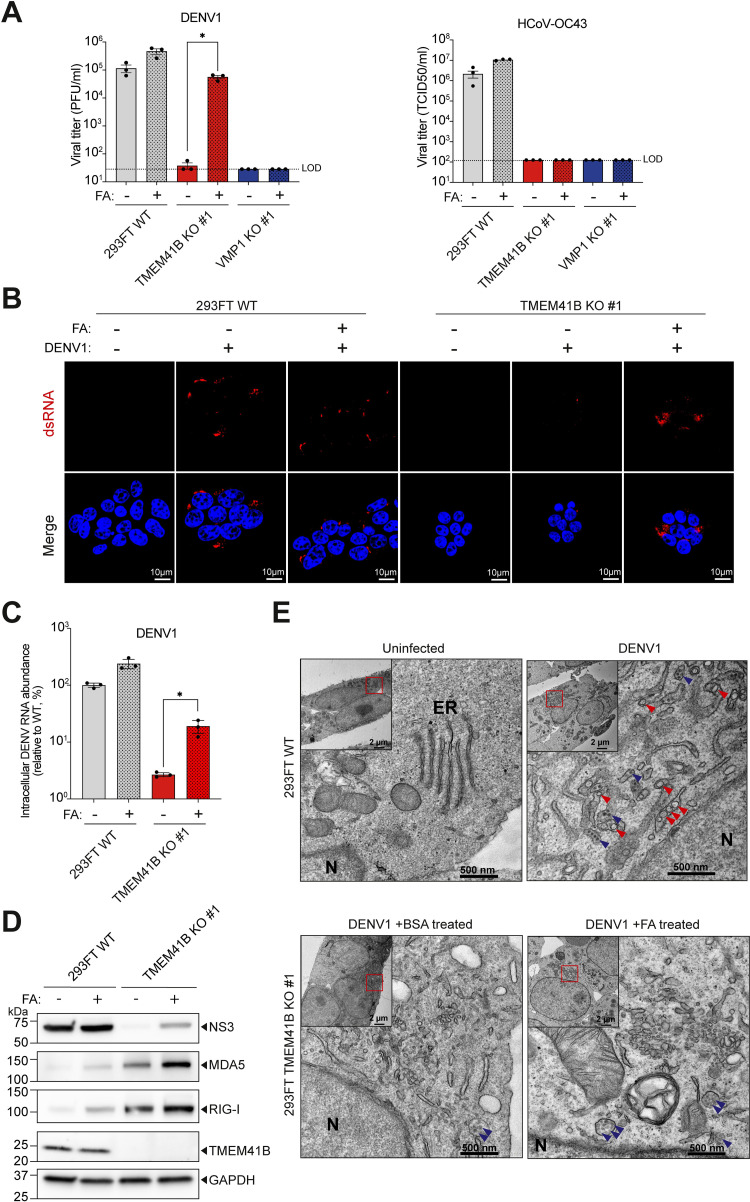
Exogenous fatty acid complementation can partially rescue DENV infection in 293FT TMEM41B KO cells but not VMP1. **(A)** Infectious viral particles produced in 293FT WT, TMEM41B KO clone #1, and VMP1 KO clone #1 cells upon FA supplementation. DENV1 infection was carried out at MOI of 1 and progeny viruses were harvested at 72 hours post-infection. HCoV-OC43 infection was done at MOI of 0.5 and progeny viruses were harvested at 48 hours post-infection. Cells were treated with a 1:1 mixture of oleic and linoleic acid (FA), or BSA. **(B)** Confocal microscopy imaging of WT and TMEM41B KO clone #1 cells infected with DENV1 and supplemented with FAs or BSA; dsRNA is stained in red and cell nuclei are displayed in blue. Image scales are indicated in the merged images. **(C)** RT-qPCR analysis of DENV RNA levels in infected (MOI of 1, 48 hours post-infection) WT and KO cells, upon treatment with FA or BSA. Values demonstrated are relative to DENV RNA load in infected WT cells. 18S rRNA has been used as the internal control. **(D)** Western blotting analysis to compare DENV viral protein accumulation in WT and TMEM41B KO cells, with and without FA supplementation. Cells were infected with DENV1 at MOI of 1 and lysates were harvested 48 hours post-infection. GAPDH was used as a loading control. **(E)** TEM imaging of DENV1-infected (MOI of 5, 48 hours post-infection) 293FT WT and TMEM41B KO clone #1. Virion-like particles and classical DENV ROs are indicated with blue and red arrowheads, respectively. Nuclei are identified by “N”. The ER structures are indicated as “ER”. Scale bars are as indicated. All data shown represent results from at least two independent experiments. Error bars represent mean +/- SEM, n = 3. * indicates *p-value* < 0.05 as determined by two-tailed unpaired t-test or one-way ANOVA. LOD indicates the limit of detection.

Both TMEM41B- and VMP1-deficient cells were previously reported to exhibit enlarged lipid droplets. While this observation has led to the unraveling of TMEM41B’s role in mobilizing FAs from lipid droplets [16], whether VMP1 can function analogously is unknown. Interestingly, DENV is known to induce lipophagy in the infected cells, facilitating the release of FAs from lipid droplets to the mitochondria [27]. Therefore, we investigated whether the impact of TMEM41B and VMP1 deficiencies could be rescued by treating the clonal KO cells with exogenous FAs. Upon DENV infection, we supplemented the WT, TMEM41B KO, and VMP1 KO 293FT cells with the mono-unsaturated FA, oleic acid, in conjunction with the polyunsaturated FA, linoleic acid. The outcome was determined by measuring released progeny virions and visualizing the viral protein accumulation in the infected cells. Surprisingly, we observed ~2–3 logs of restoration in DENV progeny virion production from two clonal TMEM41B-deficient cells treated with FAs, indicating that the genetic deficiency can be metabolically rescued (Figs 3A and S3A and S3B). Consistently, the partial restoration of DENV replication in TMEM41B KO cells upon FAs treatment could also be visualized by elevated viral RNA (Figs 3B and 3C and S3C) and protein accumulation (Fig 3D). However, FA supplementation failed to rescue DENV infection in VMP1 KO cells, suggesting that the genetic deficiency cannot be chemically salvaged or that VMP1’s potential role in lipid mobilization is not involved in DENV replication (Figs 3A and S3A and S3B). Intriguingly, the FA treatment could not rescue HCoV-OC43 infection in both TMEM41B- and VMP1-deficient cells (Fig 3A), hinting that deprived cellular FAs in these clonal KO cells might not solely be responsible for diminished viral infection. While DENV infection could be restored in TMEM41B KO cells upon FA treatment, this supplementation did not reduce RIG-I and MDA5 levels, reinforcing that the partial restoration of DENV infection was not due to suppression of RIG-I and MDA5 activity (Fig 3D). Therefore, we utilized transmission electron microscopy (TEM) to further characterize the status of DENV replication in TMEM41B KO cells. Notably, while we could identify the formation of classical DENV replication organelles (ROs) [32] in all DENV infected WT cells under the designated conditions, such structures were undetectable in DENV infected TMEM41B KO cells regardless of FA supplementation (Figs 3E and S3C). Instead, some enlarged, fragmented ER-like structures resembling aberrant ROs were visualized in the infected KO cells, albeit to various degrees of size and extensiveness. On average, these abnormal structures were considerably more convoluted in KO cells supplemented with FAs (Figs 3E and S3C). All these observations suggest that while FAs can partially rescue DENV RNA replication and progeny virion production, defective DENV replication organelles may still be present, which consequently leads to the induction of dsRNA sensors.

### TMEM41B and VMP1 deficiencies compromise mitochondrial beta-oxidation in 293FT cells

**Fig 4:**
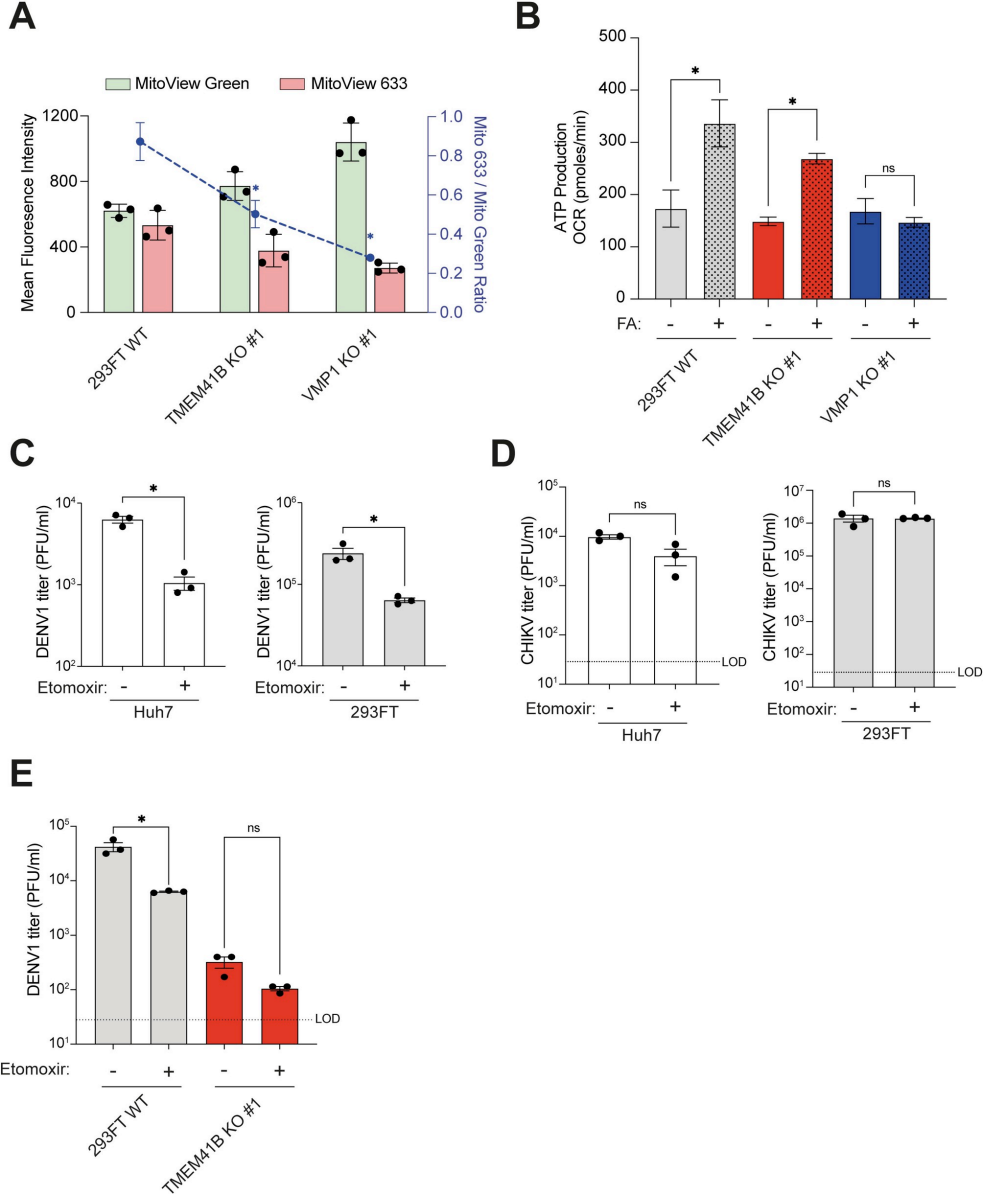
Impaired mitochondrial beta-oxidation contributes to reduced DENV infection in 293FT TMEM41B KO and VMP1 KO cells. **(A)** Comparison of total (MitoView Green) vs functionally active (MitoView 633) mitochondria level in 293 FT WT, TMEM41B KO clone #1, and VMP1 KO clone #1 cells. Blue dashed line indicates the ratio of MitoView 633 to MitoView Green signal level. **(B)** Beta-oxidation associated ATP production in the WT and clonal KO cells upon FA supplementation, measured by extracellular flux analysis. **(C)** Impact of Etomoxir treatment on production of infectious DENV particles in 293FT WT and Huh7 WT cells. Cells were infected with DENV1 at MOI of 1 and treated with 200 μM Etomoxir for 48 hours. **(D)** Impact of Etomoxir treatment on production of infectious CHIKV particles in 293FT WT and Huh7 WT cells. Cells were infected with CHIKV at MOI of 0.1 and treated with 200 μM Etomoxir for 24 hours. **(E)** Impact of Etomoxir treatment on production of infectious DENV particles in 293FT WT cells and TMEM41B KO clone #1. Cells were infected with DENV1 at MOI of 1 and treated with 200 μM Etomoxir for 48 hours. All data shown represent results from at least two independent experiments. Error bars represent mean +/- SEM, n = 3. * indicates *p-value* < 0.05 as determined by two-tailed unpaired t-test or one-way ANOVA. LOD indicates the limit of detection.

Most FAs released from lipid droplets are devoured into mitochondrial beta-oxidation under normal physiological conditions [37]. Moretti *et al*. have previously reported a slight yet significant decrease in beta-oxidation level in their TMEM41B KO cells, likely due to reduced FAs released from lipid droplets [16]. Moreover, neuronal VMP1-deficient cells were recently demonstrated to harbor morphologically impaired mitochondria [38], which might impact their beta-oxidation capacity. Since it was previously highlighted that FA metabolism and the ATP produced through mitochondrial beta-oxidation are crucial for DENV replication [27,39], we decided to assess mitochondria’s functionality and beta-oxidation capacity in TMEM41B- and VMP1-deficient cells. We first measured the ratio of functionally active mitochondria in the WT and KO cells using MitoView mitochondrial staining. MitoView staining permits the comparison of total mitochondrial mass (MitoView Green) and mitochondria with functional membrane potential (MitoView 633) in live cells as an indicator of mitochondria activity. In comparison to WT cells, we observed a significant reduction in the proportion of functional mitochondria in both KO cell lines, with a more severe mitochondrial dysfunction observed in VMP1-deficient cells (Fig 4A). Of note, the number of active mitochondria was lesser in VMP1 KO cells compared to WT cells despite a significant increase in the overall number of mitochondria (Fig 4A). The observation implied an accumulation of dysfunctional mitochondria in VMP1 KO cells, which is consistent with a recent report, demonstrating that VMP1 is also required to recycle malfunctional mitochondria through mitophagy [40]. In alignment with the lower levels of active mitochondria observed in VMP1 KO cells, we parallelly detected a marked reduction of basal cellular respiration in these KO cells (S4A and S4B Fig). Since FA treatment failed to rescue DENV infection in VMP1 KO cells, we decided to test whether this was due to impaired beta-oxidation capacity. We thus supplemented FAs to glucose-starved TMEM41B KO and VMP1 KO cells and subsequently measured their beta-oxidation-linked ATP production using the Seahorse metabolic flux assay. Interestingly, while TMEM41B KO cells exhibited the ability to utilize supplemented FAs for boosting ATP production, we could not detect the same in VMP1 KO cells (Fig 4B). The discrepancy between both KO cell lines suggested that VMP1-deficient cells exhibit a more compromised beta-oxidation capacity, which may be due to more pronounced damages to mitochondria.

To further examine the role of beta-oxidation in DENV replication, we chemically perturbed 293FT and human hepatoma Huh7 WT cells using a well-known beta-oxidation inhibitor, Etomoxir. In line with a previous report [41], we observed a significant reduction in infectious DENV viral particles production upon Etomoxir treatment, emphasizing the importance of beta-oxidation for DENV (Fig 4C). Of note, Etomoxir did not exhibit any significant inhibitory impact on CHIKV infection (Fig 4D), in agreement with the fact that TMEM41B and VMP1 are dispensable for CHIKV infection (Fig 1E). No significant cytotoxicity effects were also observed (S4C Fig), ruling out the concerns over the potential inhibition of cell growth that indirectly impacts DENV infection. Additionally, Etomoxir treatment did not lead to a significant reduction of DENV infection in TMEM41B KO cells, suggesting that the genetic and chemical perturbations could not synergistically block DENV infection (Fig 4E). Nonetheless, the magnitude of the reduction of DENV infection in WT cells caused by the Etomoxir perturbation was not comparable to the substantially diminished levels observed in TMEM41B and VMP1 deficiencies. Collectively, these results suggested that the compromised mitochondrial beta-oxidation activity detected in the KO cells is one of the factors contributing to the diminished DENV replication, but some other underlying mechanisms are likely to impact the viral infection in parallel.

### TMEM41B and VMP1 exhibit a broad yet distinct impact on the cellular metabolomic landscape in 293FT cells

**Fig 5:**
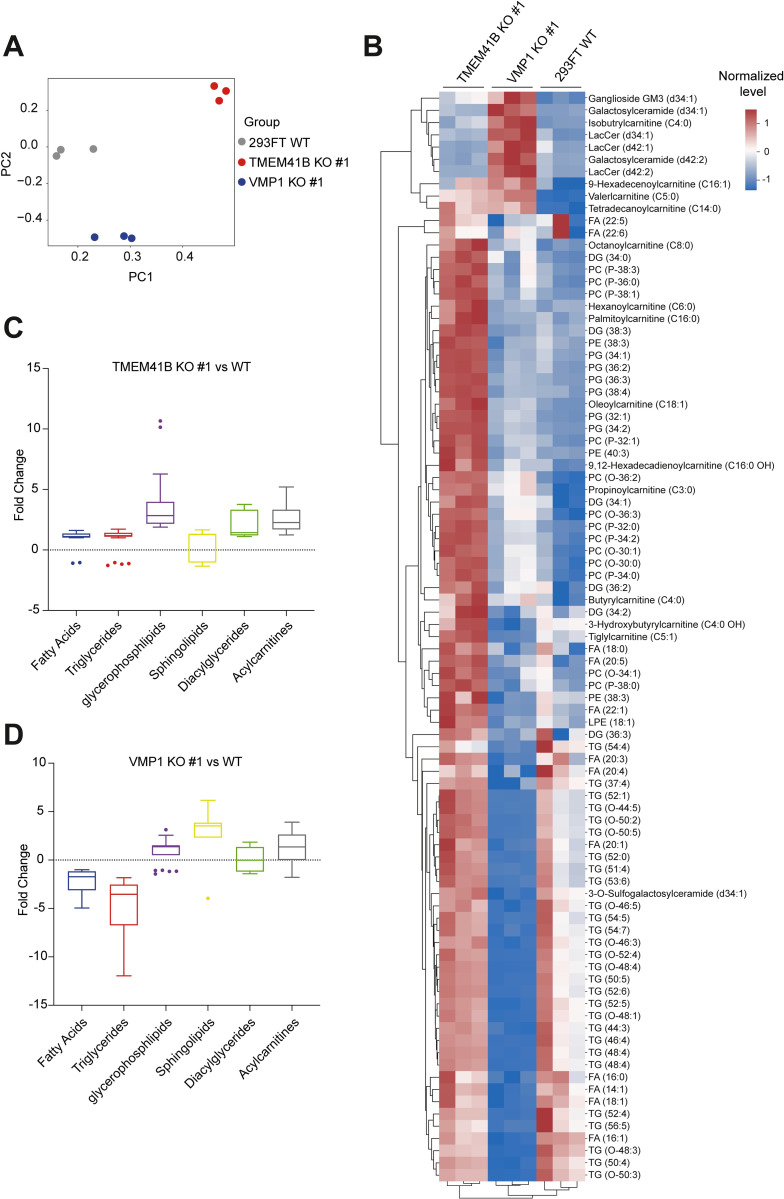
TMEM41B and VMP1 deficiency impose a diverse and distinctive impact on cellular metabolism in 293FT. **(A)** Principal Components Analysis (PCA) for the metabolomic datasets of 293FT WT, TMEM41B clone #1, and VMP1 KO clone #1 cells. First two principal components are demonstrated on the x- and y-axis. **(B)** Heatmap of altered metabolites in the 293FT WT and KO clones. Z-score normalized values of metabolite levels were used to plot the heatmap (see Materials and Methods). Color scheme depicts the relative abundance of metabolites with red and blue indicating higher and lower levels, respectively. Columns indicate different samples, each with 3 replicates. **(C)** Relative fold changes of metabolites level in TMEM41B KO clone #1 cells in comparison to 293FT WT. Values associated with metabolites from the same family are grouped together in each boxplot. Negative values represent metabolites with decreased abundance in the KO cells, as described in the Materials and Methods. **(D)** Relative fold changes of metabolites level in VMP1 KO #1 cells in comparison to 293FT WT. Values associated with metabolites from the same family are grouped together in each boxplot. Negative values represent metabolites with decreased abundance in the KO cells, as described in the Materials and Methods.

VMP1 and TMEM41B have been associated with multiple metabolic pathways, suggesting that the absence of these ER proteins may exert an extensive impact on cellular metabolism, which may explain their crucial role in the viral permissiveness of cells [18–20,42,43]. To gain insight into these global-scale metabolic impacts, we subjected the uninfected 293FT WT, clonal TMEM41B KO, and clonal VMP1 KO cells to mass spectrometry for metabolome profiling. Indeed, as indicated by the Principal Component Analysis (PCA), we observed a very clear distinction in the overall metabolic profiles of WT versus the KO cells (Fig 5A), marked by dysregulation of numerous lipid species upon TMEM41B and VMP1 deficiencies (Fig 5B). Remarkably, despite all the biochemical similarities these two ER scramblases shared, the profile of metabolic dysregulations due to TMEM41B deficiency was relatively distinct from the genetic ablation of VMP1. In particular, we observed an accumulation of several lipid species such as phospholipids and diacylglycerides in TMEM41B KO cells (Fig 5C), possibly due to the impaired intracellular lipid mobilization and/or full activation of lipid synthesis [16,19]. In contrast, FAs and triglycerides were largely depleted in VMP1-deficient cells, while these cells harbored a higher abundance of several sphingolipids (Fig 5D). Interestingly, we also observed massive dysregulations in a vast majority of metabolites involved in central cellular energy metabolism pathways, such as the tricarboxylic acid (TCA) cycle and glycolysis, likely attributed to the alterations in lipid metabolism and mitochondria activity in the KO cells (S5A and S5B Fig) [44]. Taken together, we revealed that TMEM41B and VMP1 deficiencies have widely derailed homeostasis of cellular lipidome and metabolome, further supporting the notion that TMEM41B and VMP1 are essential host factors for specific groups of enveloped viruses through multiple parallel mechanisms.

## Discussion

Hitherto, we understand that in addition to structural similarities, both TMEM41B and VMP1 function as phospholipid scramblases [18–21], play a crucial role in the cellular distribution of cholesterol and phospholipids [20], regulate early stages of autophagosome formation [15–17,42] and, indeed, are host dependency factors uniquely required for several enveloped viruses [11–14,28]. However, it has also been demonstrated that TMEM41B and VMP1 are not functionally redundant, and each of these two proteins is associated with other non-mutual functions and cellular pathways [38,43,45]. Whether VMP1 and TMEM41B cooperate in augmenting viral infections has yet to be elucidated. In addition, it is also unclear whether these proteins play similar roles in different viral infections.

**Fig 6:**
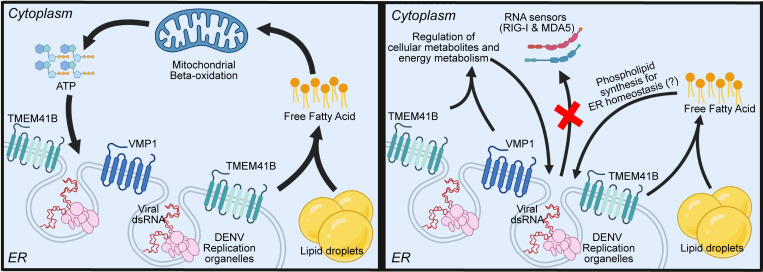
The schematic model for multiple roles of TMEM41B and VMP1 in DENV infection. TMEM41B and VMP1 modulate ER homeostasis, lipid mobilization, mitochondrial beta-oxidation, and global metabolomic regulations to facilitate productive DENV infection. This figure was created with Biorender.com.

In this study, we presented a model (Fig 6) for the roles of TMEM41B and VMP1 in DENV infection, in which multiple cellular events, such as the ER membrane dynamic, mitochondrial beta-oxidation, and cellular metabolome are involved. Notably, TMEM41B is likely to be required for mobilizing FAs to fuel metabolic pathways such as FA beta-oxidation, which is essential for productive DENV infection, while VMP1 may similarly facilitate this process by mediating proper mitochondrial functionality. The crucial role of VMP1 in beta-oxidation is also recently shown by Jiang *et al*., *in vivo*, further confirming the importance of VMP1 for maintaining the normal mitochondrial beta-oxidation [46]. On the other hand, TMEM41B and VMP1 may also fine-tune the physiological levels of numerous metabolites, including many lipid species essential for productive viral replication and biogenesis. For instance, the virulence properties of enveloped viruses might be influenced by the levels of sphingolipids and glycerophospholipids, as these lipid species are vital components of cellular membranes that constitute the envelope of the progeny viral particles [47–50]. Acylcarnitines, essential components of mitochondrial beta-oxidation, have been shown to be critical metabolites sequestered by Wolbachia to restrict DENV replication in mosquitoes [41]. Perhaps, this massive metabolic dysregulation upon TMEM41B and VMP1 deficiencies could partially explain their vital necessity in facilitating coronavirus infections as well. Interestingly, a recent report proposed that TMEM41B contributes to efficient HCoV-229E replication by regulating the cellular membrane cholesterol composition [13]. However, it remains unclear why only specific groups of enveloped RNA viruses are dependent on TMEM41B and VMP1. In the case of alphaviruses, one possible explanation lies within their replication strategy – exploiting the plasma membrane instead of the ER membrane for viral RNA synthesis and particle budding. On the contrary, flaviviruses, and coronaviruses predominantly rely on ER-derived membranes to complete their infection cycle. Further studies are needed to elucidate the molecular mechanisms by which VMP1 and TMEM41B are recruited in viral infections and how these ER membrane proteins distinctively impact different families of enveloped viruses. For instance, it is worth mentioning that TMEM41B co-localizes and interacts with non-structural proteins, NS4A and NS4B, of Zika virus and yellow fever virus [12]. Intriguingly, NS4A and NS4B have been previously reported to play a role in inducing lipophagy during DENV infection [25,39]. Thus, whether or not the interaction between TMEM41B and viral proteins plays an additional role in mediating lipid metabolism will need to be determined. Correspondingly, it has been recently demonstrated that VMP1, but not TMEM41B, can directly interact with nsp3 and nsp4 proteins of a murine beta-coronavirus [28].

Importantly, our results substantiated the possibility of aberrant flavivirus RNA replication in TMEM41B KO cells, as evidenced by the absence of classical DENV ROs. We postulate that DENV failed to replicate in VMP1 KO cells in a similar fashion, as hinted by the upregulations of RIG-I and MDA5. Nevertheless, the activation of the upstream sensors did not further generate a similar level of amplification in TBK1 activation, as well as IFN-β induction. The underlying mechanism by which RIG-I and MDA5 were upregulated in the infected KO cells is not known, but the lack of a correlation between RIG-I/MDA5 and IFN-β levels would suggest that cytokine-independent mechanisms may be involved [51,52]. Surprisingly, TBK1 was pre-activated in both TMEM41B KO and VMP1 KO cells prior to infection. The molecular mechanism leading to this basal activation is yet to be determined. Notably, depletion of RIG-I and MDA5 failed to promote DENV infection in the KO cells, indicating that the upregulation of RNA sensors is unlikely to be the main contributing factor limiting DENV replication. These results are in consonance with the report by Hoffmann *et al*., in which pharmacological inhibitions of the innate immune signaling pathway (i.e. TBK1 and JAK), were unable to restore flavivirus infections in the TMEM41B KO cells [12].

Our findings address some of the knowledge gaps by highlighting the metabolic roles of TMEM41B and VMP1 in facilitating DENV infection. However, it is important to state some of the key limitations of the experimental designs. Most of the mechanistic studies presented in this report, especially metabolomics profiling, were performed using 293FT cell line. 293FT is a clonal isolate of 293T cells [53], which permits the generation of single-cell derived CRISPR KO clones that most likely share a similar genetic background. This is also supported by our experiments showing that different KO clones of 293FT yielded similar virus infection outcomes, especially in the FA rescue assay (Figs 3 and S3B). However, it would be potentially challenging to prove the generality of these observations to other cell lines and cell types. Thereupon, our approach may not be able to pinpoint specific metabolic alterations which occur under physiological conditions, but the key message is that genetic perturbations of TMEM41B and VMP1 can lead to a broad dysregulation of cellular metabolism, especially lipid metabolism. Hence, more in-depth studies using relevant models, e.g., human primary cells, *ex vivo* culture systems, and *in vivo* models, would be crucial to pinpoint the exact metabolic dysregulation patterns in TMEM41B and VMP1 deficiencies.

Untangling the different roles of TMEM41B and VMP1 would be complex, but a comprehensive grasp of them could offer tantalizing opportunities. As host dependency factors for several clinically important RNA viruses, these ER proteins may serve as promising targets for innovating broad-spectrum antiviral strategies. Our findings together with all recent discoveries about TMEM41B and VMP1 have collectively deciphered some of the underlying molecular mechanisms that may be crucial to pave the way for developing host-directed therapeutics against flaviviruses and human coronaviruses.

## Materials and methods

### Cell lines and reagents

293FT (provided by JEC), Huh7.5.1 (provided by JEC), RD (ATCC, provided by JEC), BHK-21 (ATCC, provided by JEC) and Huh7 cells (a gift from Linfa Wang) were cultured in Dulbecco’s modified Eagle’s medium (high glucose) supplemented with 10% heat-inactivated fetal bovine serum (FBS), penicillin-streptomycin, and L-glutamine. A549 cells (ATCC), C6/36 (ATCC, provided by EEO), and Vero E6 (ATCC, provided by JEC) were cultured in RPMI-1640 supplemented with 10% FBS, penicillin-streptomycin, and L-glutamine. All cell lines were tested negative for mycoplasma.

### Viral strains and serotypes

DENV1 (NR-3782; BEI resources, provided by JEC), DENV2 (Eden3295; clinical isolate provided by EEO [54]), DENV3 (NR-44088; BEI resources, provided by JEC), DENV4 (NR-3806; BEI resources, provided by JEC), and SINV (Ar-339; ATCC, provided by JEC) were propagated on C6/36 cells. HCoV-OC43 (VR-1558; ATCC, provided by GJDS) was propagated on RD cells. CHIKV (DMERI009/08, clinical isolate provided by EEO) was propagated on BHK-21 cells. JEV (SA14-14-2; vaccine strain provided by EEO) was propagated on Vero E6.

### Generation of isogenic KO cells

Single-cell derived TMEM41B KO and VMP1 KO clones were generated using a CRISPR/Cas9 editing strategy. Firstly, guide RNA (sgRNA) sequences against *TMEM41B* and *VMP1* were derived from human CRISPR Brunello lentiviral pooled libraries (Addgene, #73178) [55], and the corresponding oligos were purchased from Integrated DNA Technologies. The oligos 5’-CACCGAGCAGTAAAATGGTCACAGC-3’ (for *TMEM41B*) and 5’-CACCGATGGCATCGTCAAAGCATTG-3’ (for *VMP1*) were cloned into the Cas9-expressing PX458 plasmid (Addgene, #48138) [56] generated by the Zhang lab. The cloning products were transfected into 293FT and A549 cells using TransIT-LT1 transfection reagent (Mirus Bio LLC, USA) according to the manufacturer’s protocol. 48 hours post-transfection, cells were single-cell sorted based on GFP marker expression into 96-well plates using a BD Influx cell sorter at the Stanford Shared FACS facility or Duke-NUS FACS facility. Colonies were expanded from single cells before being lysed for genotyping of the designated genomic regions. A roughly 500 base-pair region that encompassed the sgRNA-targeted site was amplified for genotyping, and the sequence of the PCR product was determined by Sanger sequencing. Colonies were selected in which all alleles harbored insertions or deletions that were not a factor of three (S1 Table). Genotyping primers were 5’-TACCCTCCCCACCTCTGAAA-3’ and 5’-GCACTGGGAGTTGGCTGATA-3’ (for *TMEM41B*), and 5’-GAAAAGGCTACAGTGGGGGT-3’ and 5’-CCTCAGCAGCCATAAAACATCA-3’ (for *VMP1*). Knock-out subclones verified by genotyping were further confirmed by western blotting.

### Generation of pooled CRISPR KO or cDNA expressing cell lines using 3^rd^ generation lentivirus system

For the generation of RIG-I and MDA5 KO cells, sgRNA sequences were adopted from [57] and oligos were purchased from Integrated DNA Technologies. The oligos 5’-AAACAACAAGGGCCCAATGG-3’ (for *RIG-I*) and 5’-TTGGACTCGGGAATTCGTGG-3’ (for *MDA5*) were cloned into the Cas9-expressing LentiCRISPRv2 plasmid (Addgene, #52961) [58]. Lentivirus particles were produced by co-transfection of the sgRNA cloned plasmids, with a mixture of pMDLg/pRRE, pRSV-REV, VSV-G, and pAdVAntage packaging plasmids into 293FT cells using TransIT-LT1 transfection reagent (Mirus Bio LLC). Lentiviruses from the supernatant were harvested at 48 hours post-transfection, cleared by centrifugation (500×g, 10 minutes), and were mixed before adding to 293FT cells along with 1×protamine sulfate. Transduced cells were selected by puromycin treatment (2 μg/ml, InvivoGen) to enrich for RIG-I and MDA5 knocked-out cells. An empty LentiCRISPRv2 plasmid was used to package lentivirus for transducing the cells to control for the lentivirus transduction effects.

For the generation of cDNA complemented cell lines, *TMEM41B* cDNA and *VMP1* cDNA were PCR retrieved from pMRXIP TMEM41B-3xFLAG (Addgene, #117415) and pMRXIP 3xFLAG-VMP1 (Addgene; #117416) [15] using primer oligos 5’-TGTGGTGGAATTCTGCAGATACCATGGCGAAAGGCAG-3’ and 5’-CGGCCGCCACTGTGCTGGATTTACTCAAATTTCTGCT-3’ (for *TMEM41B*), and 5’-TGTGGTGGAATTCTGCAGATACCATGGCAGAGAATGG-3’ and 5’-CGGCCGCCACTGTGCTGGATTTATTTAGTTTTCTCCTC-3’ (for *VMP1*). The amplified PCR products were gel purified and cloned into the EcoRV-digested pLenti-CMV-Puro-Dest (w118-1) (Addgene; #17452) [59] using NEBuilder HiFi DNA Assembly (New England Biolabs) according to the manufacturer’s protocol. The cloning products were packaged into lentivirus particles as described above and used to transduce KO cells. A lentivirus carrying the mCherry cDNA [57] was used to transduce KO cells to control for the lentivirus transduction effects.

### Cell growth kinetics

Cells were seeded on 24-well cell culture plates, and total cell numbers were counted at 24, 48 and 72 hours post-seeding using trypan blue staining and an automated cell counter (Bio-Rad). Cells doubling time per day were calculated by comparing the initial and final population count (Roth V. 2006 Doubling Time Computing) [60].

### Virus infection and titration

Cells were seeded one day prior to infection on 24-well cell culture plates and were infected the next day at the indicated multiplicity of infections (MOIs) for all infection assays. Cells were incubated with the virus inoculum for 3 hours before replacing it with fresh media. Progeny viruses from the supernatant were harvested at the indicated time points. DENV and CHIKV viral titers were assessed by plaque assays on Huh7.5.1 cells, using 1–1.5% methylcellulose (Sigma) and 1.5% Avicel (Sigma) overlays, for DENV and CHIKV respectively. Plates were fixed using 4% paraformaldehyde (Sigma) at 6 days post-infection for DENV1 and DENV4, 3 days for CHIKV, and 7 and 8 days for DENV2 and DENV3, respectively. Fixed plates were washed and stained with crystal violet. HCoV-OC43 viral titers were determined by the end-point titration in RD cells. Cells were inoculated with fivefold serial dilutions of virus and incubated for 6 days, before scoring the number of wells displaying cytopathic effects. The values of the 50% Tissue Culture Infectious Dose (TCID_50_) per ml were calculated using the Spearman-Kärber method.

### Crystal violet staining

293FT WT, TMEM41B KO and VMP1 KO cells were seeded in 96-well cell culture plates and were subsequently infected with DENV1, DENV2, JEV, CHIKV, and SINV at MOI of 10. For HCoV-OC43, cells were infected at MOI of 2. Cells infected with DENV1 and DENV2 were fixed using 4% paraformaldehyde at 7 days post-infection, all other cells were fixed at 5 days. Cell viability at the time of fixation was determined by standard crystal violet staining.

### Western blotting and antibodies

For harvesting cells lysates, cells were first washed with cold phosphate buffer (PBS) and then lysed with 1× RIPA buffer (Sigma) containing 1× protease inhibitors cocktail (Nacalai Tesque). 4× Laemmli buffer (Bio-Rad) supplemented with 10% β-mercaptoethanol (Sigma) was added to the lysates and then boiled for 10 min. Samples were separated by SDS-PAGE on pre-cast 4–20% polyacrylamide Mini-PROTEAN gels (Bio-Rad) and transferred onto nitrocellulose or PVDF membranes (Bio-Rad). The membranes were blocked with a 5% blotting-grade blocker (Bio-Rad) dissolved in 1× PBS (Axil Scientific) containing 0.1% Tween-20 (Sigma). The blocked membranes were subsequently washed and incubated overnight with a primary antibody diluted in the blocking buffer at 4°C on a rocker. All images were taken using a Bio-Rad Chemidoc Touch machine and analyzed by Image Lab software (Bio-Rad). The following primary antibodies were used to detect the presence of indicated proteins in this study: TMEM41B (HPA014946; 1:700; Sigma-Aldrich), VMP1 (12978S; 1:1000; Cell Signaling Technology), GAPDH (GTX627408; 1:5000; GeneTex) or (60004-1-Ig; 1:10000; Proteintech), DENV NS3 (GTX124252; 1:3000; GeneTex), HCoV-OC43 N (MAB9012; 1:3000; Merck), RIG-I (3743S; 1:1000; Cell Signaling Technology), MDA5 (5321S; 1:1000; Cell Signaling Technology), TBK1 (3504S; 1:1000; Cell Signaling Technology), and p-TBK1 (5483S; 1:1000; Cell Signaling Technology).

### RT-qPCR

Cells were lysed and cDNA was directly synthesized from total RNA using a Power SYBR Green Cells-to-C_T_ Kit (Invitrogen), according to the manufacturer’s protocol. Quantitative PCR (qPCR) was performed using a Bio-Rad CFX96 machine. Primer pairs employed include: *IFNB1* (forward: 5’-ATGACCAACAAGTGTCTCCTCC-3’, reverse: 5’-GGAATCCAAGCAAGTTGTAGCTC-3’); *18S ribosomal RNA* (forward: 5’-AGAAACGGCTACCACATCCA-3’, reverse: 5’-CACCAGACTTGCCCTCCA-3’); and DENV viral RNA (forward: 5’-GGTTAGAGGAGACCCCTCCC-3’, reverse: 5’- GGTCTCCTCTAACCTCTAGTCC-3’) as previously described [7,57]. C_T_ values were normalized to 18S rRNA levels for each sample.

### Lipid complementation and β-oxidation drug treatments

Cells were seeded on 24-well cell culture plates and were incubated overnight at 37°C. Virus inoculum was added to the cells at the designated MOI the next day. Cells were incubated with the virus inoculum for 3 hours before substitution with fresh media supplemented with lipids or small molecules. For lipid complementation, a 1:1 mixture of BSA-conjugated oleic acid (Sigma) and BSA-Conjugated linoleic acid (Sigma) were added to the cells, each at 2.5 mg.ml^-1^ concentration (~75 μM FA). Media supplemented with 5 mg.ml^-1^ BSA (Sigma) was added to control samples. For beta-oxidation drug experiments, cells were incubated with DMEM complete media containing 200 μM Etomoxir (Sigma), as reported by Manokaran *et al*. [41]. Progeny viruses released in the supernatant were harvested at 48 hours post-infection and titered by plaque assay as described above.

### Confocal imaging

293FT WT, TMEM41B KO, and VMP1 KO cells were seeded on cover glasses coated with poly-D-Lysine (Sigma) and incubated overnight at 37°C. The next day, cells were infected with DENV1 virus inoculum at MOI of 1. At 3 hours post-infection the inoculum was replaced with media supplemented with FAs or BSA as described above and the cells were fixed with 4% PFA (Thermo Fisher Scientic) at 16 hours post-infection. Cells were washed and incubated with anti-dsRNA primary antibody (MABE1134; 1:100; Merck) for 2 hours. Cells were incubated with secondary antibody (A-11005; 1:1000; Invitrogen) and Hoechst (H3570; 1:10000; Invitrogen) for 45 min and were subsequently washed and fixed on slides. Images were taken at the Duke-NUS Medical School imaging facility using a Zeiss LSM710 confocal microscope at 100× of magnification. Images depicted in Fig 3B represent more than 20 cells showing similar observations in each condition. The anti-dsRNA rJ2 antibody signal could be detected by eye in 31/45 (~68.9%) of BSA-treated and 35/41 (~85.4%) of FA-treated WT cells. The signal was observed in ~18.9% (7/37) of BSA-treated TMEM41B KO cells and was increased to ~57.7% (30/52) upon FA treatment. Total dsRNA intensity for each image was quantified using “MeasureImageIntensity” function of the CellProfiler software (www.cellprofiler.org) [61,62]. Mean dsRNA signal intensity per cell was calculated by dividing the total intensity values by the number of cells captured in each image, as indicated by Hoechst staining.

### TEM imaging

Briefly, 5x10^6^ 293FT WT and TMEM41B KO clone #1 cells were infected with DENV1 at MOI of 5 PFU/cell. FA complementation of KO cells was carried out as described above. The cells were fixed at 48 hours post-infection using 2.5% Glutaraldehyde (Ted Pella, Inc.) in PBS for 4 hours. The samples were then washed 3 times in PBS, and treated with 1% Osmium tetroxide (Ted Pella, Inc.) and 1.5% potassium ferrocyanide for 1 hour. Next, the samples were washed in deionized water before harvesting cells by scrapping. Cell pellets were dehydrated in ethanol and acetone, followed by infiltration and embedding in Araldite 502 resin. Samples were sectioned using Leica EM UC6 to produce ultrathin sections. Ultrathin sections were stained using lead citrate. All TEM images were taken using JEM 1400 Flash (JEOL LTD, Japan) at 100kV. More than 30 single cells were examined for each sample and images from at least 10–15 intact cells exhibiting the presence of DENV classical/abnormal RO structures were captured per condition, in two different magnifications. A DENV particle was characterized as a 30–60 nm spherical electron-dense object, which was enclosed within the ER-like structures. Classical DENV ROs appeared as spherical, enlarged ER-like structures, enclosed within the ER-derived membranes [32].

### Assessment of total mitochondria and mitochondria potential

Mitoview mitochondrial dyes (Biotium), the Mitoview Green (1:2500), and Mitoview 633 (1:750) dyes were used to stain for total mitochondria and membrane potential respectively. After incubation with cells for 30 min at 37°C, cells were trypsinized and washed twice with MEM maintenance media. Flow cytometry was performed at Duke-NUS FACS facility using BD Fortessa FACS analyzer and the mean fluorescence intensity (MFI) was measured and analyzed using FlowJo v10. MFI of MitoView 633 was taken as a ratio against MFI of MitoView Green to assess the overall function of mitochondria within the cells.

### Seahorse mito stress test

293FT WT, TMEM41B KO, and VMP1 KO cells were seeded in seahorse XF-24 cell culture microplates (Agilent) at 1x10^5^ cells per well. The next day, mitochondria stress test was performed using the seahorse XFe24 Analyzer (Agilent) platform, using the protocol recommended by Agilent. The step-by-step protocol has been previously described [63]. Briefly, the drugs, oligomycin (final concentration 1 μM), carbonyl cyanide-4-(trifluoromethoxy) phenylhydrazone (FCCP) (1.5 μM), rotenone (100 mM), and antimycin-A (1 μM) were added sequentially as per the protocol. All the drugs listed were purchased from Sigma. Readouts of the mitochondria stress test were analyzed using Seahorse Wave software (Agilent). Basal oxygen consumption rate (OCR) is defined as the initial OCR before the addition of inhibitors minus the non-mitochondrial respiration; ATP-linked OCR is obtained after the addition of oligomycin which inhibits ATP synthase (Complex V).

For measurement of mitochondrial bioenergetics with FA supplementation, experimental conditions were modified as follows: Two days prior to the measurement, cells were seeded and incubated at 37°C overnight. The next day, the cells were starved by exchanging growth media with substrate-limited media supplemented with linoleic and oleic acid or BSA as control (Seahorse XF RPMI medium, pH 7.4 containing 1mM glutamine, 1% FCS, 2.5 mg.ml^-1^ linoleic and oleic acid or BSA) and incubated overnight. On the day of measurement, the cells were washed three times and incubated with assay media (Seahorse XF RPMI medium, pH 7.4 plus 2.5 mg.ml^-1^ linoleic and oleic acid or BSA as control). Mito stress test was carried out as above.

### Mass spectrometry-based lipidomics and metabolomics

Lipid and metabolite extraction and lipidomics/metabolomics analyses followed the published reports with modifications [64–66]. Briefly, 1x10^8^ 293FT, TMEM41B KO and VMP1 KO cells were washed with PBS thrice before adding 560 μl extraction solvent (methanol:water = 2:5), pre-cooled at 4°C. Cells were then scraped into the extraction solvent on ice and transferred into Eppendorf tubes. Then, 800 μL methyl tert-butyl ether (MTBE) was added and the samples were sonicated for 10 min. After centrifugation for 15 min at 3,000 rpm at 4°C to separate phases, the upper layer and lower layer were collected for lipidomics and metabolomics analysis, respectively.

The untargeted lipidomics analyses were performed with Agilent 1290 ultrahigh pressure liquid chromatography system equipped with a 6550 QTOF mass detector equipped with a dual-spray electrospray ionization source with Jet Stream (Agilent Technologies, Santa Clara, CA). The column used for the separation was an Agilent rapid resolution HT Zorbax SB-C18 (2.1´100 mm, 1.8 mm). The oven temperature was set at 45°C. The gradient elution involved a mobile phase consisting of (A) ACN-water (60:40) (B) ACN-IPA (10:90), both containing 10mM ammonium formate with 0.1% formic acid. The initial condition was set at 40% B for the first 2 min, followed by a 12 min gradient to 100% B which was held for 3 min, then returned to starting conditions over 0.5 min. Flow rate was set at 0.4 ml/min, and 5 μL of samples was injected. The electrospray ionization mass spectra were acquired in both positive and negative ion mode. Mass data were collected between m/z 100 and 1000 at a rate of two scans per second. The ion spray voltage was set at 4,000 V, and the heated capillary temperature was maintained at 350°C. The drying gas and nebulizer nitrogen gas flow rates were 12.0 L/min and 50 psi, respectively. Two reference masses were continuously infused to the system to allow constant mass correction during the run: m/z 121.0509 (C_5_H_4_N_4_) and m/z 922.0098 (C_18_H_18_O_6_N_3_P_3_F_24_). Raw spectrometric data were analyzed by MassHunter Qualitative Analysis software (Agilent Technologies, US) and the molecular features characterized by retention time, chromatographic peak intensity and accurate mass, were obtained by using the Molecular Feature Extractor algorithm. Only features with an intensity ≥ 20,000 counts (approximately three times the limit of detection of our LC-MS instrument), and found in at least 80% of the samples at the same sampling time point signal were kept for further processing. Next, a tolerance window of 0.15 min and 2 mDa was used for alignment of RT and *m/z* values. Lipid identities were assigned based on accurate mass measurement and MS/MS fragmentation patterns.

The targeted metabolomics analyses were performed with Agilent 1290 ultrahigh pressure liquid chromatography system coupled to a 6490 Triple Quadrupole mass spectrometer equipped with a dual-spray electrospray ionization source with Jet Stream (Agilent Technologies, Santa Clara, CA). Chromatographic separation of glycolysis intermediates and organic acid was achieved by using Phenomenex (Torrance, CA) Rezex ROA-Organic Acid H+ (8%) column (2.1×100 mm, 3 μm) and the compounds were eluted at 40°C with an isocratic flow rate of 0.3 mL/min of 0.1% formic acid in water. All the metabolites were quantified in multiple reaction monitoring (MRM) mode, and mass transition and collision energy were optimized for each compound by direct infusion of individual standard solutions. Electrospray ionization was performed in negative ion mode with the following source parameters: drying gas temperature 300°C with a flow of 10 L/min, nebulizer gas pressure 40 psi, sheath gas temperature 350°C with a flow of 11 L/min, nozzle voltage 500 V, and capillary voltage 3,000 V. Data acquisition and processing were performed using MassHunter software (Agilent Technologies, US).

PCA analysis was performed using prcomp function of the stats package in R. Clustergrams were generated by the clustermap function of the seaborn package in python3, using Z-score normalized values of all samples for each metabolite separately, with mean = 0 and SD = 1. Data table values used to generate the heatmaps are provided in S2 Table. The scripts used to generate the clustergarams can be accessed from https://github.com/kuanrongchan/TMEM41B-VMP1-KO. For boxplots showing metabolites’ relative levels, average values of the three replicates were used to calculate the ratio in respect to WT samples. For the metabolites with decreased levels in the KO cells (Ratio: x<1) we have substituted the ratio with “– 1/x” for visualization purposes. Raw mass spectrometry data prior to analysis are uploaded to Metabolomics Workbench [67] with Study ID ST002164 (http://dev.metabolomicsworkbench.org:22222/data/DRCCMetadata.php?Mode=Study&StudyID=ST002164&Access=JrgV9574).

### Statistical analysis

All statistical analyses were performed by GraphPad Prism v9 (GraphPad software). *P-values* were calculated using appropriate statistical testing including a two-tailed unpaired t-test and one-way ANOVA.

## Supporting information

S1 Fig cDNA complementation of TMEM41B KO and VMP1 KO cells. **(A)** Western blotting analysis of DENV2 NS3 accumulation in 293FT WT, TMEM41B KO clone #2, VMP1 KO clone #2 and their cDNA-complemented cells. Cells were infected with DENV2 at MOI of 0.1 for 48 hours. GAPDH was used as a loading control. **(B)** Cell growth kinetics in WT, KO, and cDNA-complemented cells in 293FT (left) and A549 (right). Error bars represent mean +/- SEM, n = 6. The significance of differences in cell doubling times per day was tested using one-way ANOVA. * indicates p-value < 0.05. All data shown represent results from at least two independent experiments.
(TIF)

S2 Fig Upregulation of dsRNA sensors in TMEM41B KO and VMP1 KO cells upon DENV infection. Western blotting analysis to detect RIG-I and MDA5 protein levels in A549 WT, TMEM41B KO clone #1, and VMP1 KO clone #1 cells, upon DENV infection. Cells were infected with DENV1 at MOI of 0.5 and lysates were harvested 32 hours post-infection. GAPDH was used as a loading control. Data shown represent results from three independent experiments.
(TIF)

S3 Fig Impaired mitochondrial beta-oxidation in TMEM41B KO and VMP1 KO cells contributes to the reduced DENV infection. **(A)** Infectious viral particles produced upon FA supplementation in (left) 293FT WT, TMEM41B KO clone #1, and VMP1 KO clone #1 cells infected with DENV2, (right) 293FT WT, TMEM41B KO clone #2, and VMP1 KO clone #2 cells infected with DENV1. DENV1 and DENV2 infection was carried out at MOI of 1 and progeny viruses were harvested at 72 hours post-infection. Error bars represent mean +/- SEM, n = 3. LOD indicates the limit of detection. All data shown represent results from at least two independent experiments. **(B)** Quantification for confocal microscopy imaging of 293FTWT and TMEM41B KO clone #1 cells infected with DENV1 and supplemented with FAs or BSA. dsRNA antibody signal per cell has been calculated for each condition. Error bars represent mean +/- SEM, for at least 20 cells in each condition. **(C)** TEM imaging of DENV1-infected (MOI of 5, 48 hours post-infection) 293FT WT and TMEM41B KO clone #1. Images shown are representative of 10–15 single-cell cross-sections. Virion-like particles and classical DENV ROs are indicated with blue and red arrowheads, respectively. Nuclei are identified by “N”. The ER structures are indicated as “ER”. Scale bars are as indicated.
(TIF)

S4 Fig VMP1 KO cells exhibit decreased basal level of cellular respiration. **(A)** The seahorse assay plot of 293FT WT, TMEM41B KO clone #1, and VMP1 KO clone #1 cells cultured in glucose-rich media. Error bars represent mean +/- SD, n = 4. **(B)** Oxygen consumption rate (OCR) levels attributed to basal respiration in WT and KO cells. **(C)** Cytotoxicity of Etomoxir on 293FT and Huh7 cells. Cells were treated with either 200μM Etomoxir or equivalent dH2O one day post seeding; and were harvested to count with trypan blue 48 hours after treatment. All data shown represent results from at least two independent experiments. Error bars represent mean +/- SEM, n = 3. * indicates p-value < 0.05, as calculated by a two-tailed t-test or one way ANOVA.
(TIF)

S5 Fig Metabolites involved in glycolysis and TCA cycle exhibiting dysregulated levels upon TMEM41B and VMP1 deficiencies. **(A)** Schematic diagram of glycolysis (Top) and TCA cycle (Bottom) principal metabolites. Relative cellular levels of metabolites in 293FT WT, TMEM41B KO clone #1 and VMP1 KO clone #1 cells are plotted on right. Error bars represent mean +/- SEM, n = 3. * indicates p-value < 0.05 adjusted for multiple comparisons by two-stage Benjamini, Krieger, & Yekutie testing. **(B)** Heatmap of glycolysis and TCA cycle associated metabolites with altered levels in 293FT WT and KO cells. Z-score normalized values of metabolite levels were used to plot the heatmap as described in the materials and methods. Color scheme depicts the relative abundance of metabolites with red and blue indicating higher and lower levels respectively. Rows indicate different samples, each with 3 replicates.
(TIF)

S1 Table Genotyping results of WT and clonal KO cells generated in this study. (XLSX)

S2 Table Data tables for metabolomics results, identifying metabolites with alteration in TMEM41B KO and VMP1 KO cells. (XLSX)

S3 Table Data tables containing all the values used to generate plots. (XLSX)

S1 Data Raw WB Images. PDF File containing all the original western blotting images used to generate figure panels in this study.
(PDF)
