# Supplementary figures and images for: TMEM41B and VMP1 modulate cellular lipid and energy metabolism for facilitating dengue virus infection
Source: PLoS Pathog. 2022 Aug 8;18(8):e1010763. doi: 10.1371/journal.ppat.1010763 (PMC9387935; doi:10.1371/journal.ppat.1010763)

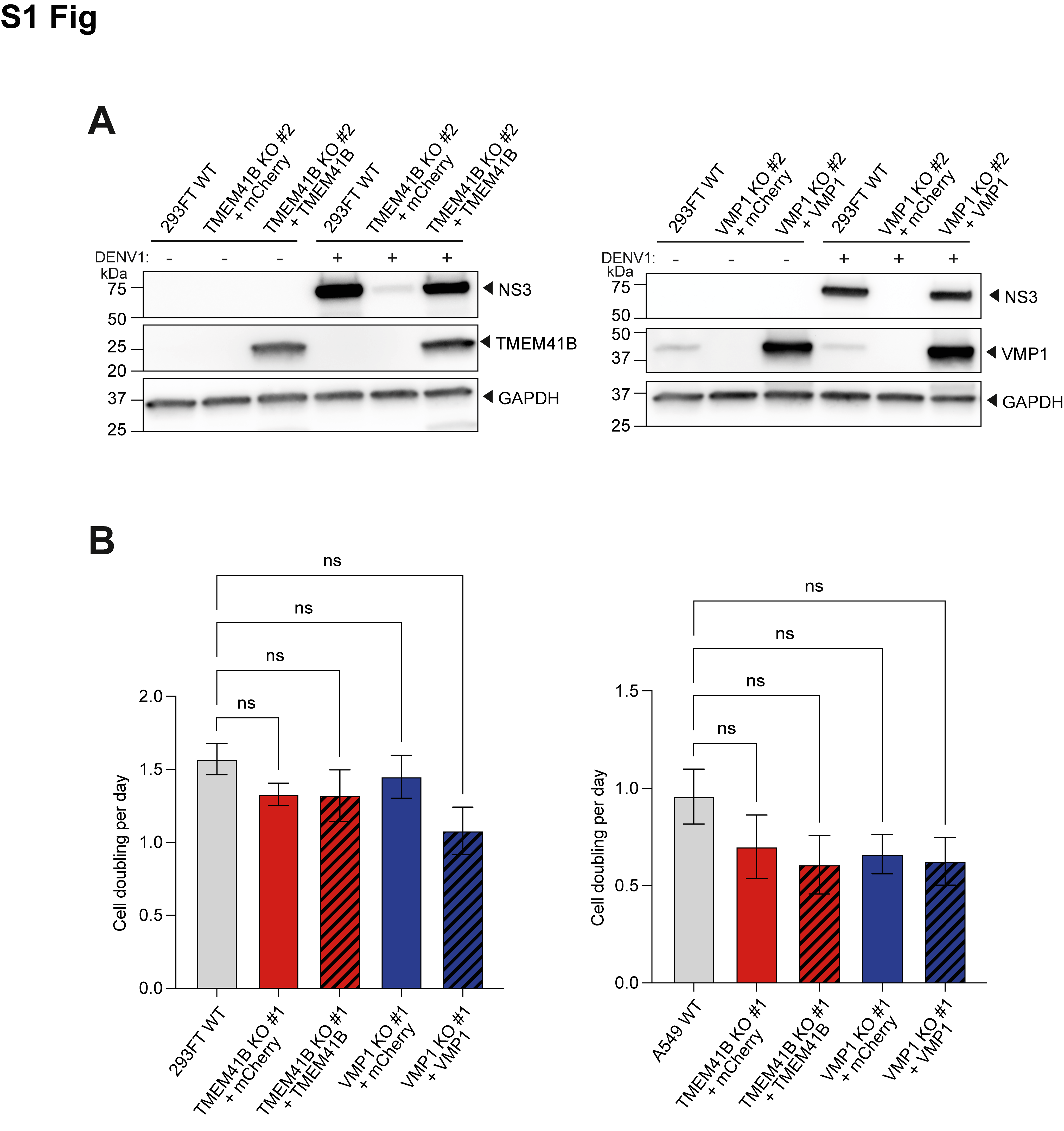

Supplement: S1 Fig — (A) Western blotting analysis of DENV2 NS3 accumulation in 293FT WT, TMEM41B KO clone #2, VMP1 KO clone #2 and their cDNA-complemented cells. Cells were infected with DENV2 at MOI of 0.1 for 48 hours. GAPDH was used as a loading control. (B) Cell growth kinetics in WT, KO, and cDNA-complemented cells in 293FT (left) and A549 (right). Error bars represent mean +/- SEM, n = 6. The significance of differences in cell doubling times per day was tested using one-way ANOVA. * indicates p-value < 0.05. All data shown represent results from at least two independent experiments. (TIF) [file ppat.1010763.s001.tif]

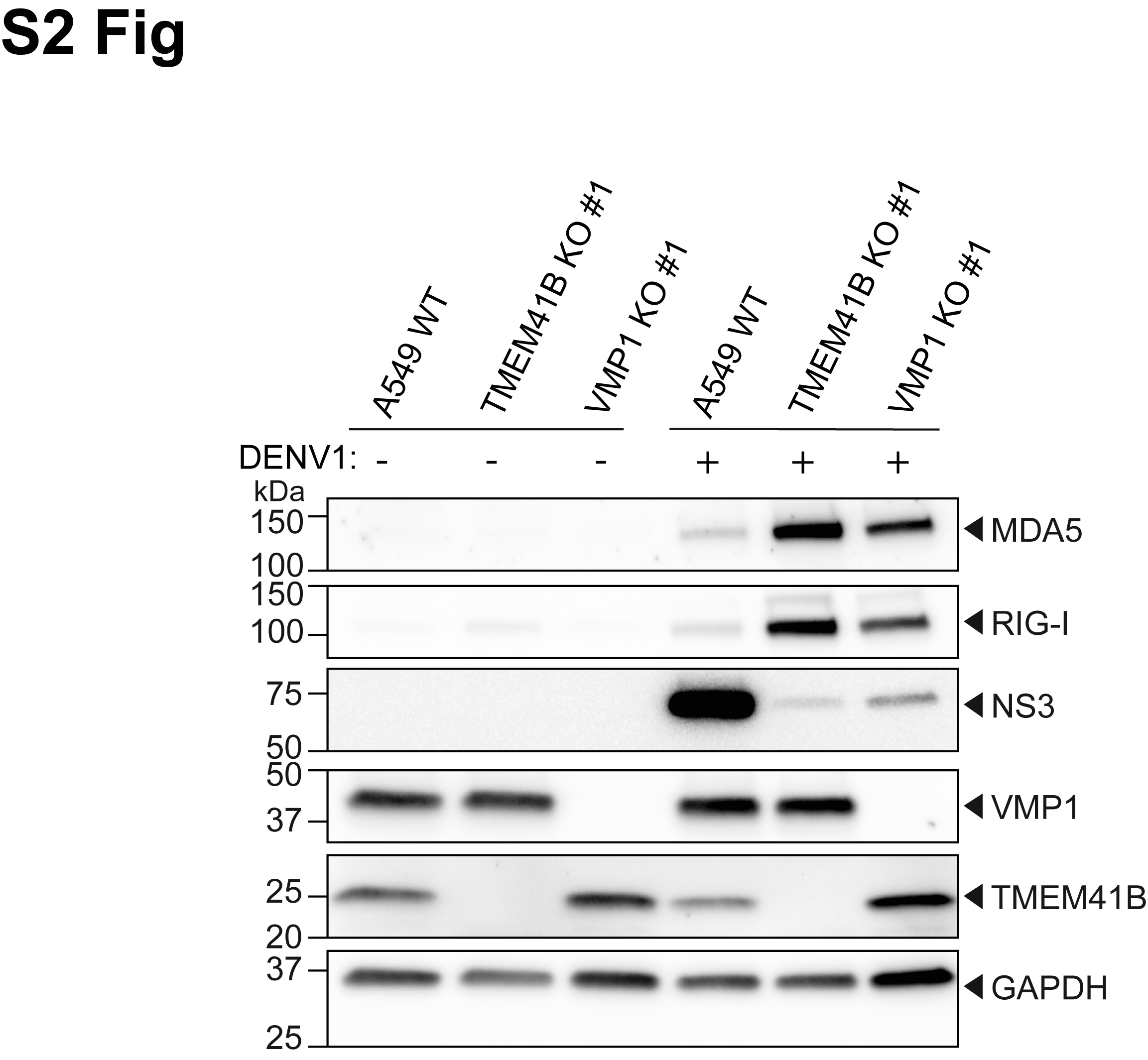

Supplement: S2 Fig — Western blotting analysis to detect RIG-I and MDA5 protein levels in A549 WT, TMEM41B KO clone #1, and VMP1 KO clone #1 cells, upon DENV infection. Cells were infected with DENV1 at MOI of 0.5 and lysates were harvested 32 hours post-infection. GAPDH was used as a loading control. Data shown represent results from three independent experiments. (TIF) [file ppat.1010763.s002.tif]

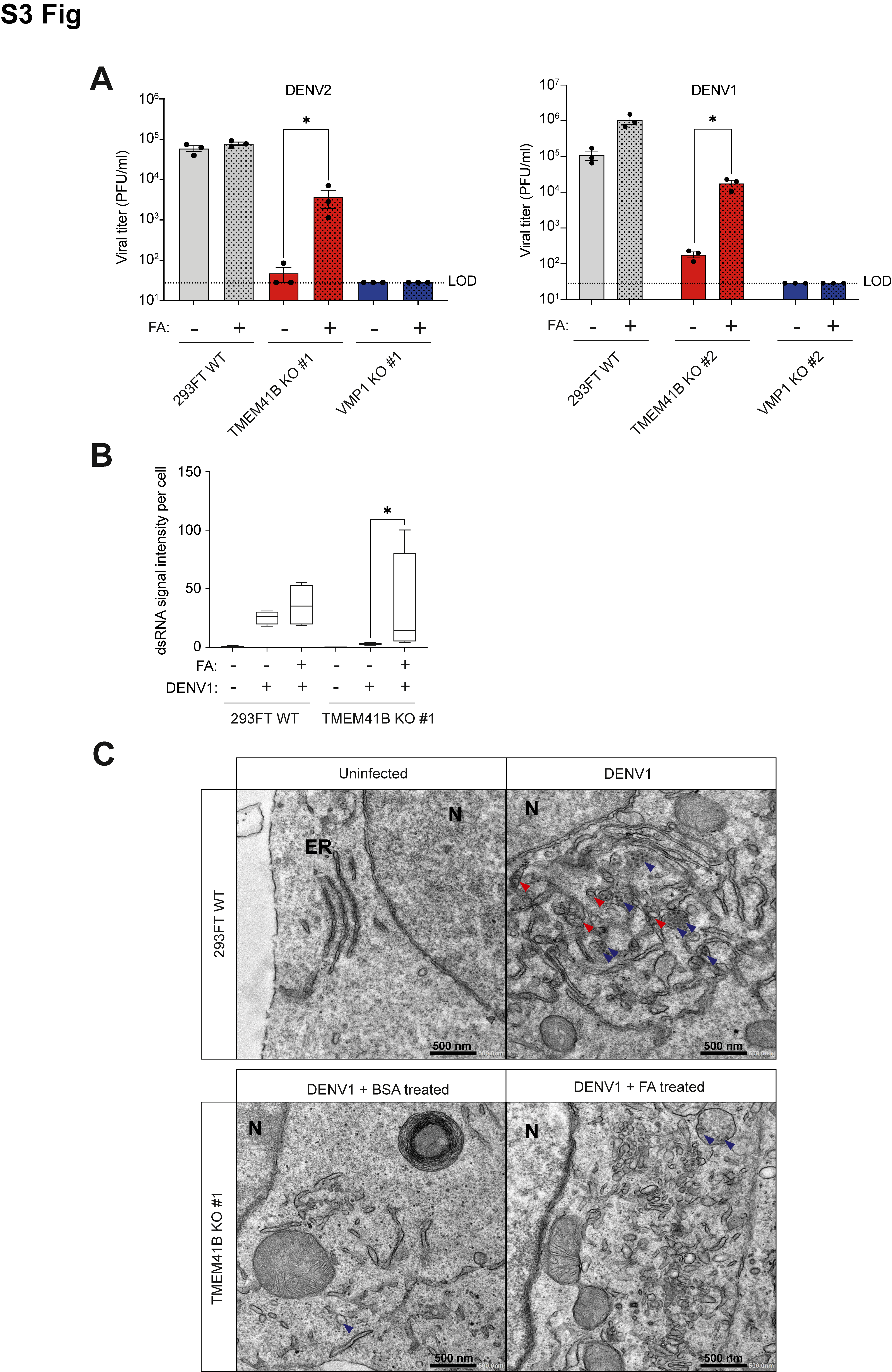

Supplement: S3 Fig — (A) Infectious viral particles produced upon FA supplementation in (left) 293FT WT, TMEM41B KO clone #1, and VMP1 KO clone #1 cells infected with DENV2, (right) 293FT WT, TMEM41B KO clone #2, and VMP1 KO clone #2 cells infected with DENV1. DENV1 and DENV2 infection was carried out at MOI of 1 and progeny viruses were harvested at 72 hours post-infection. Error bars represent mean +/- SEM, n = 3. LOD indicates the limit of detection. All data shown represent results from at least two independent experiments. (B) Quantification for confocal microscopy imaging of 293FTWT and TMEM41B KO clone #1 cells infected with DENV1 and supplemented with FAs or BSA. dsRNA antibody signal per cell has been calculated for each condition. Error bars represent mean +/- SEM, for at least 20 cells in each condition. (C) TEM imaging of DENV1-infected (MOI of 5, 48 hours post-infection) 293FT WT and TMEM41B KO clone #1. Images shown are representative of 10–15 single-cell cross-sections. Virion-like particles and classical DENV ROs are indicated with blue and red arrowheads, respectively. Nuclei are identified by “N”. The ER structures are indicated as “ER”. Scale bars are as indicated. (TIF) [file ppat.1010763.s003.tif]

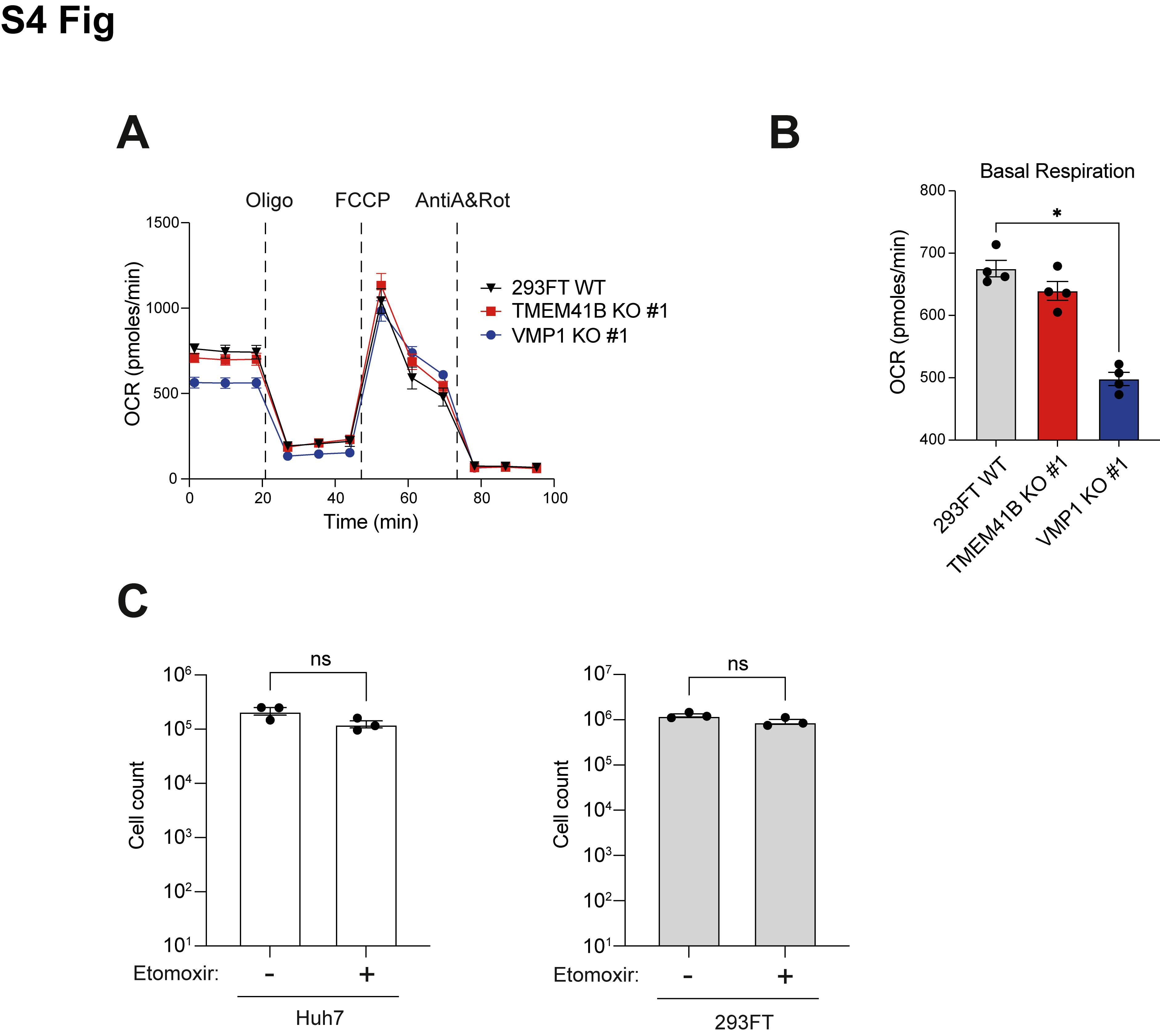

Supplement: S4 Fig — (A) The seahorse assay plot of 293FT WT, TMEM41B KO clone #1, and VMP1 KO clone #1 cells cultured in glucose-rich media. Error bars represent mean +/- SD, n = 4. (B) Oxygen consumption rate (OCR) levels attributed to basal respiration in WT and KO cells. (C) Cytotoxicity of Etomoxir on 293FT and Huh7 cells. Cells were treated with either 200μM Etomoxir or equivalent dH2O one day post seeding; and were harvested to count with trypan blue 48 hours after treatment. All data shown represent results from at least two independent experiments. Error bars represent mean +/- SEM, n = 3. * indicates p-value < 0.05, as calculated by a two-tailed t-test or one way ANOVA. (TIF) [file ppat.1010763.s004.tif]

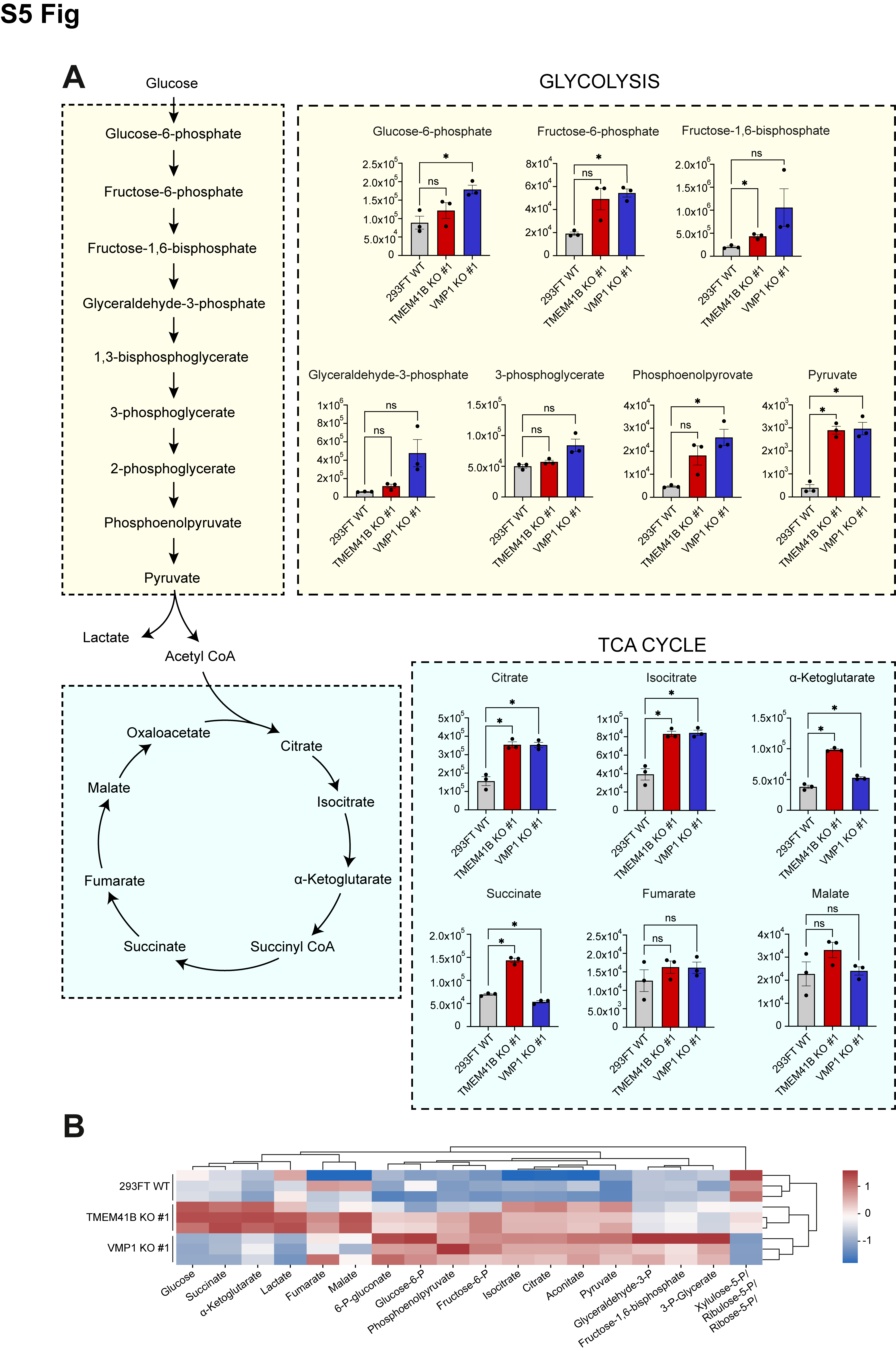

Supplement: S5 Fig — (A) Schematic diagram of glycolysis (Top) and TCA cycle (Bottom) principal metabolites. Relative cellular levels of metabolites in 293FT WT, TMEM41B KO clone #1 and VMP1 KO clone #1 cells are plotted on right. Error bars represent mean +/- SEM, n = 3. * indicates p-value < 0.05 adjusted for multiple comparisons by two-stage Benjamini, Krieger, & Yekutie testing. (B) Heatmap of glycolysis and TCA cycle associated metabolites with altered levels in 293FT WT and KO cells. Z-score normalized values of metabolite levels were used to plot the heatmap as described in the materials and methods. Color scheme depicts the relative abundance of metabolites with red and blue indicating higher and lower levels respectively. Rows indicate different samples, each with 3 replicates. (TIF) [file ppat.1010763.s005.tif]
