# Supplementary material for: TMEM41B and VMP1 modulate cellular lipid and energy metabolism for facilitating dengue virus infection
Source: PLoS Pathog. 2022 Aug 8;18(8):e1010763. doi: 10.1371/journal.ppat.1010763 (PMC9387935; doi:10.1371/journal.ppat.1010763)
Supplement: S1 Data — PDF File containing all the original western blotting images used to generate figure panels in this study. (PDF) [file ppat.1010763.s009.pdf]

Figure 1A

anti-VMP1

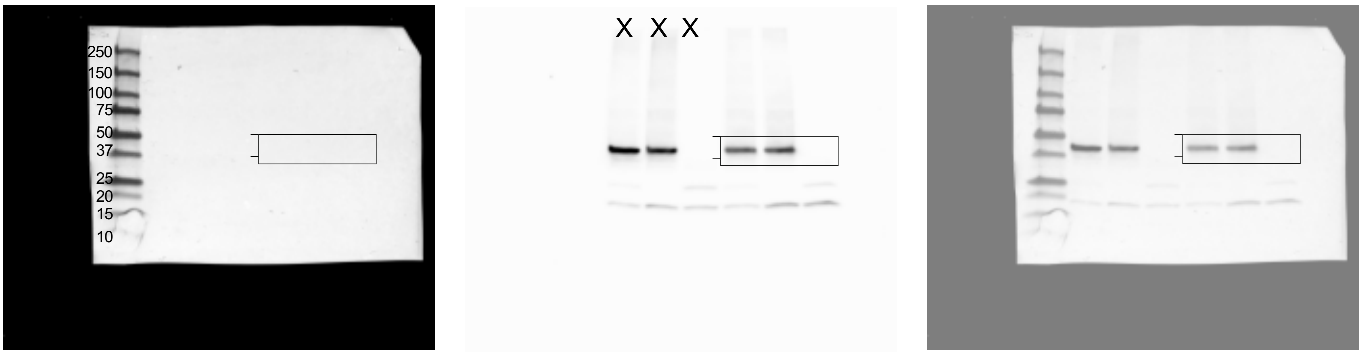

anti-TMEM41B

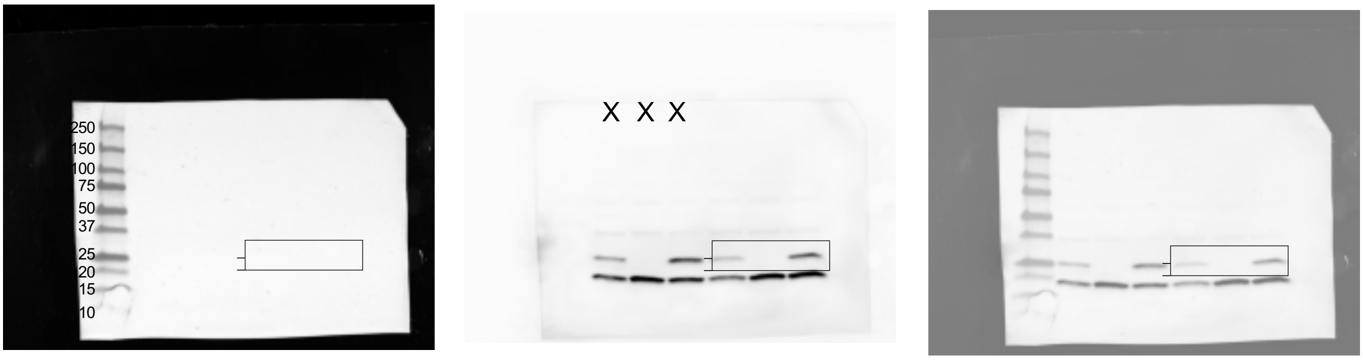

anti-GAPDH

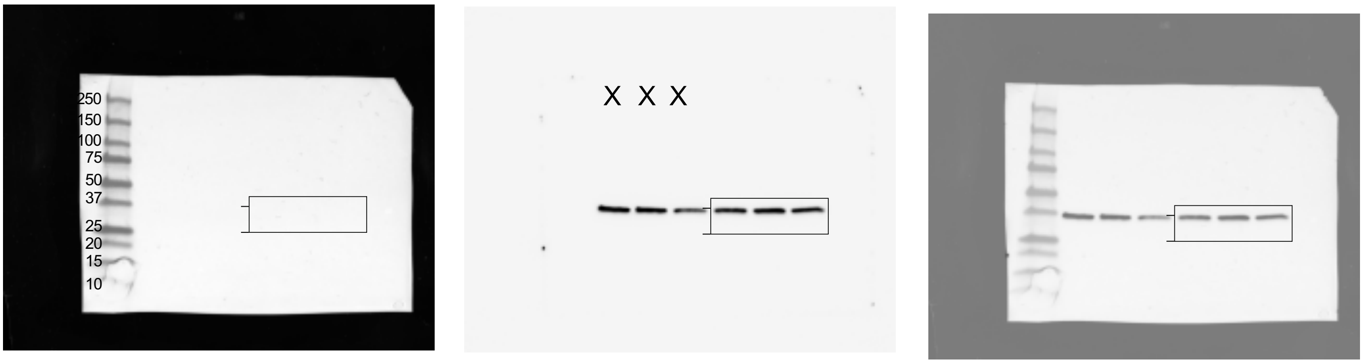

Figure 1B

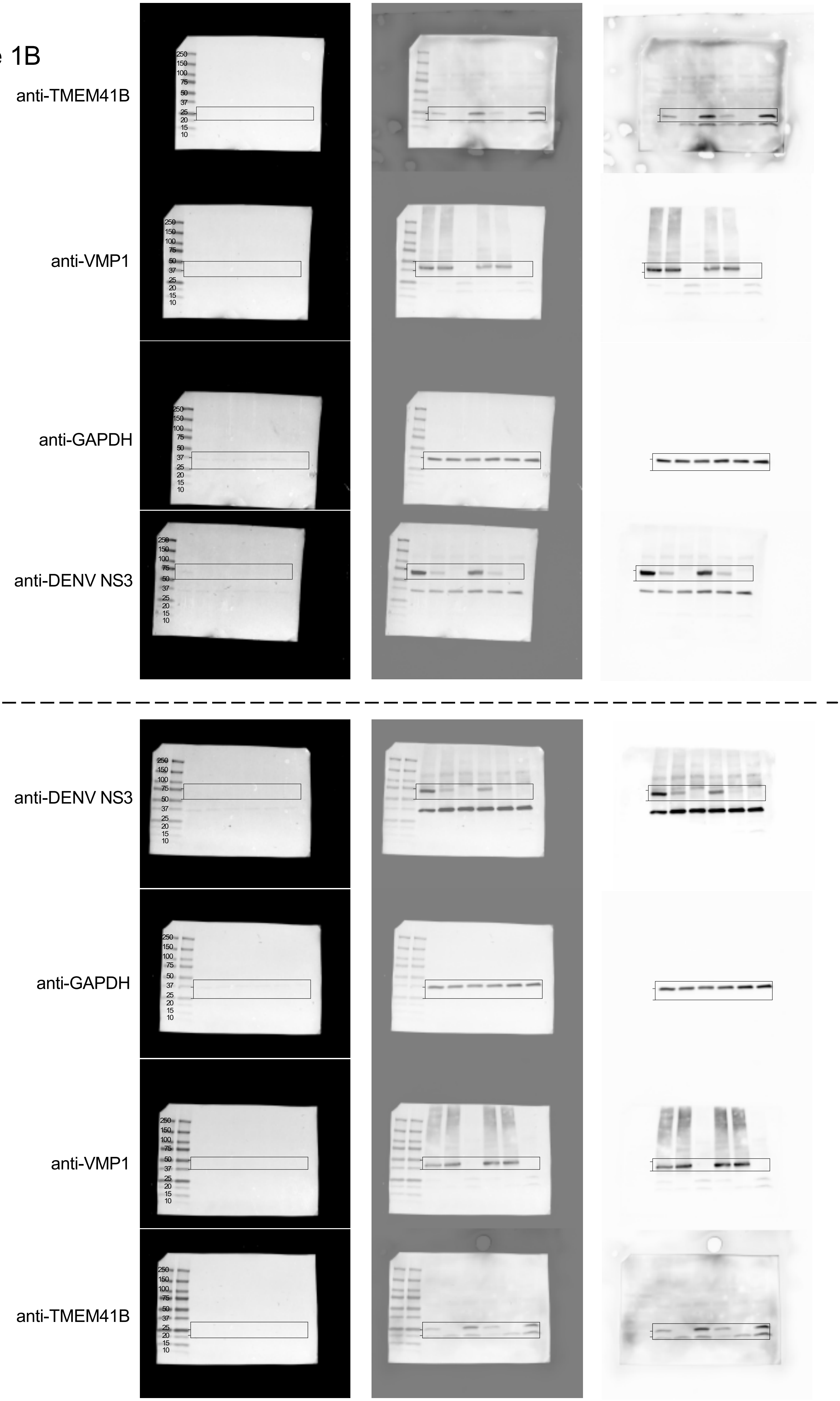

anti-TMEM41B
anti-VMP1
anti-GAPDH
anti-DENV NS3
anti-DENV NS3
anti-GAPDH
anti-VMP1
anti-TMEM41B

Figure 1D

anti-TMEM41B

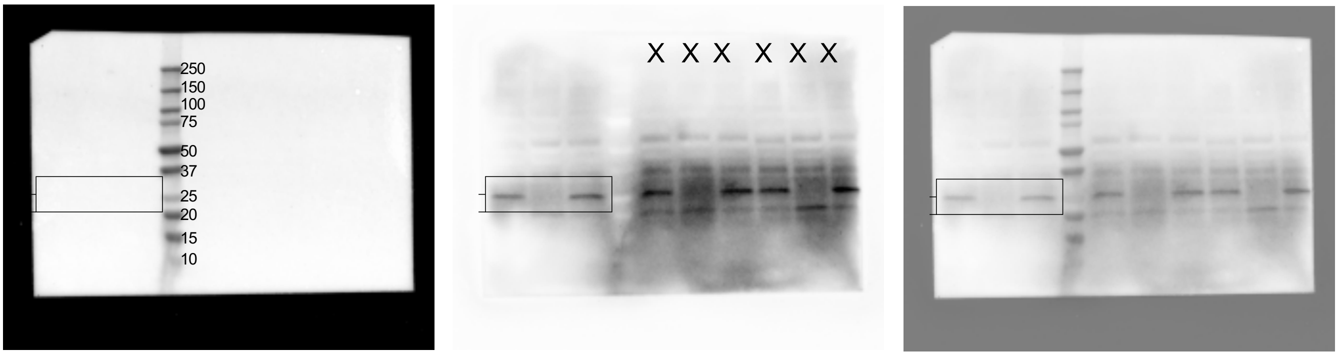

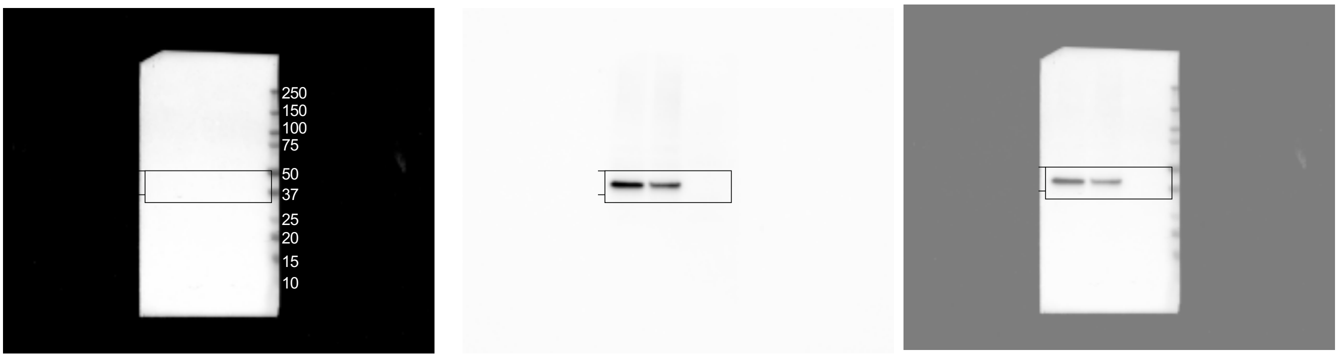

anti-VMP1

anti-OC43 N

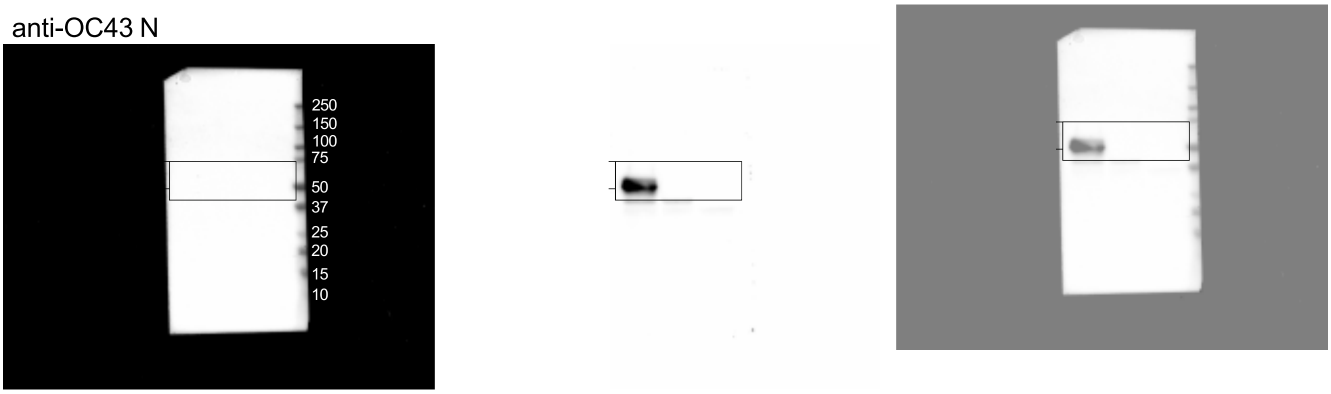

anti-GAPDH

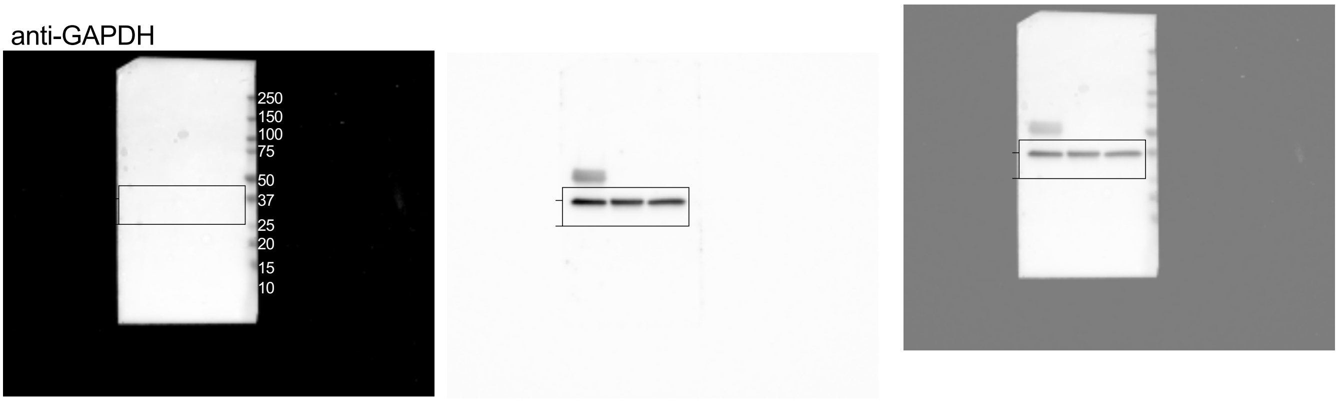

Figure 1F

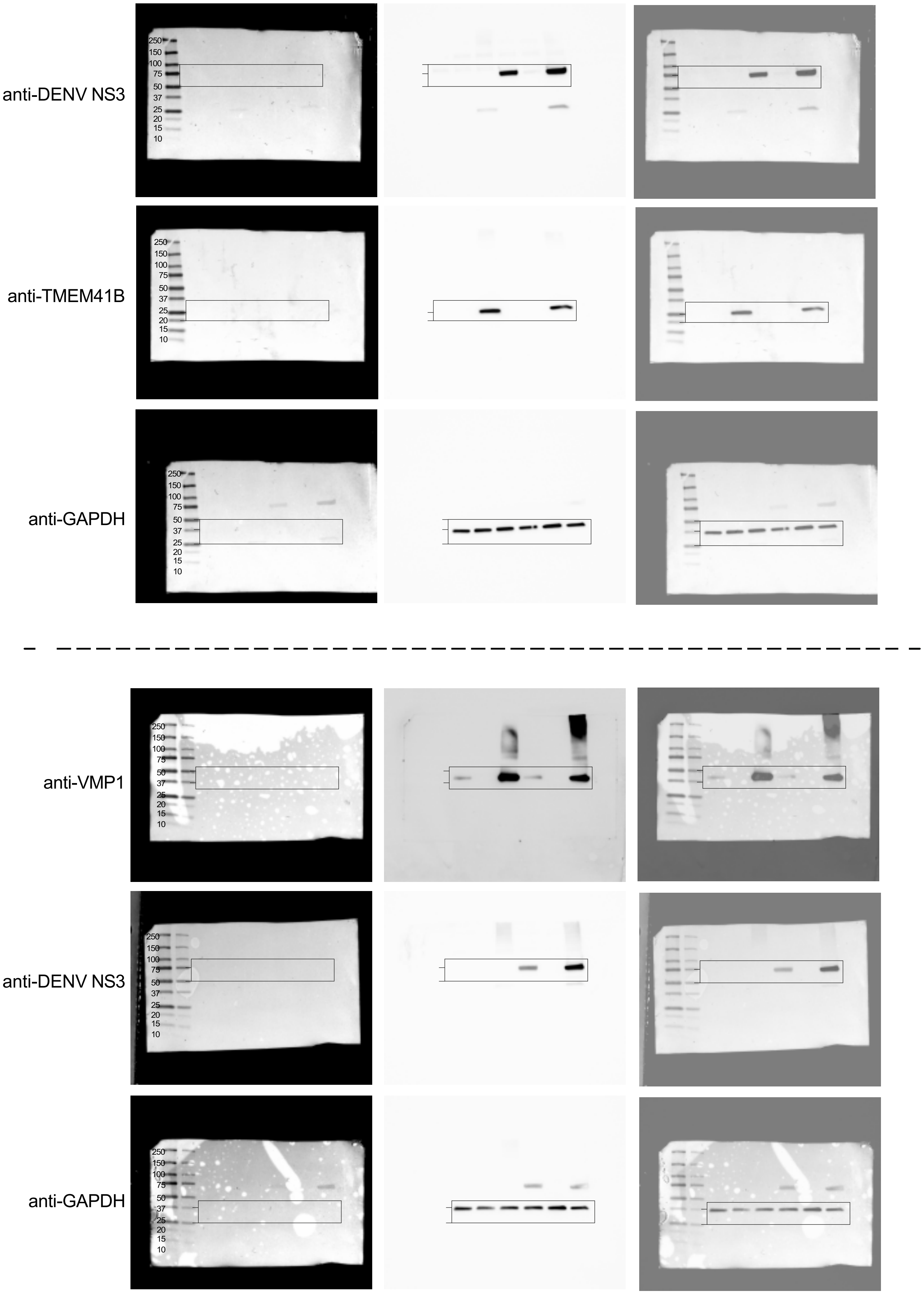

anti-DENV NS3
anti-TMEM41B
anti-GAPDH
anti-VMP1
anti-DENV NS3
anti-GAPDH
250
150
100
75
50
37
25
20
15
10

Figure 1G

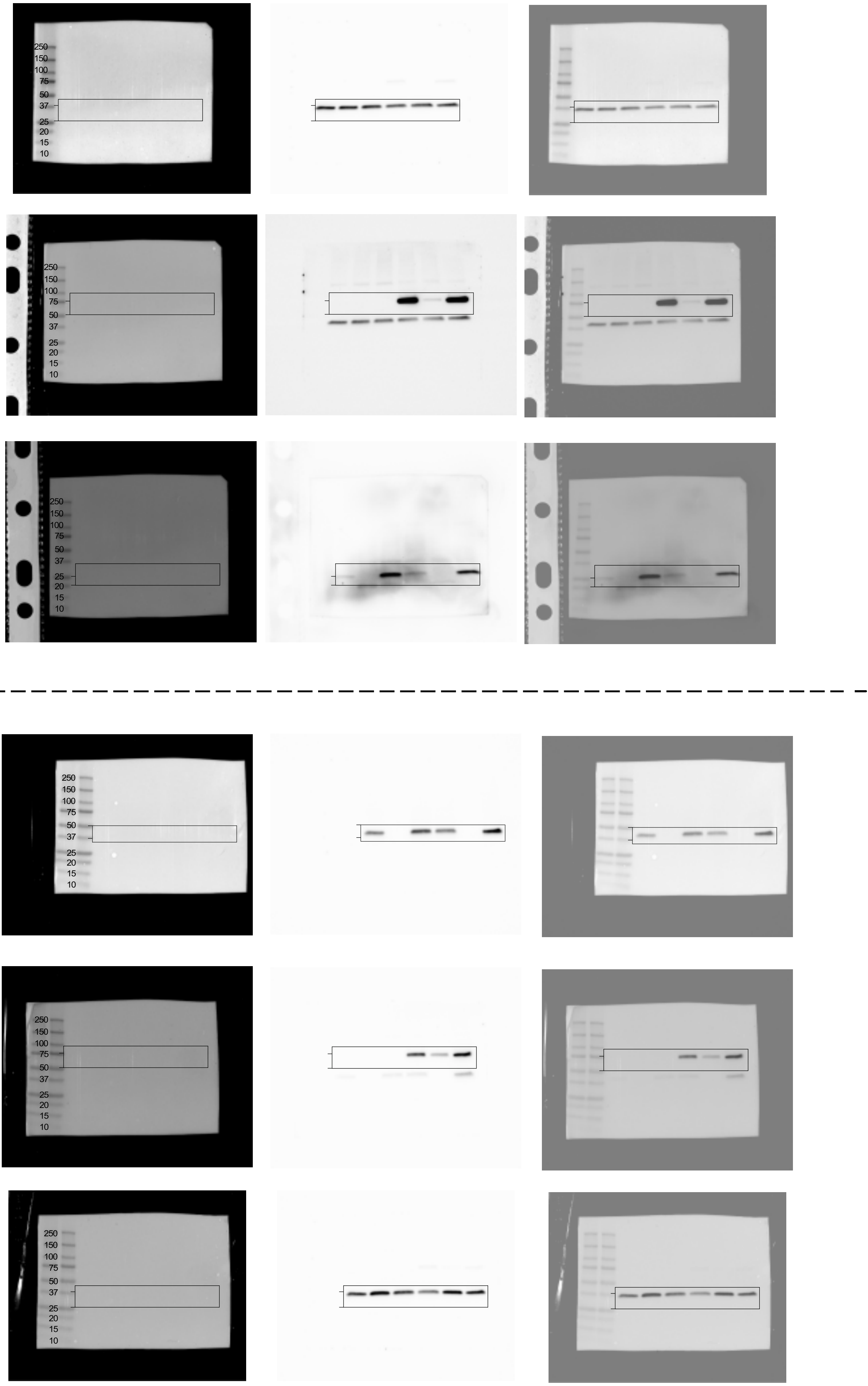

anti-GAPDH

anti-DENV NS3

anti-TMEM41B

anti-VMP1

anti-DENV NS3

anti-GAPDH

Figure 2A

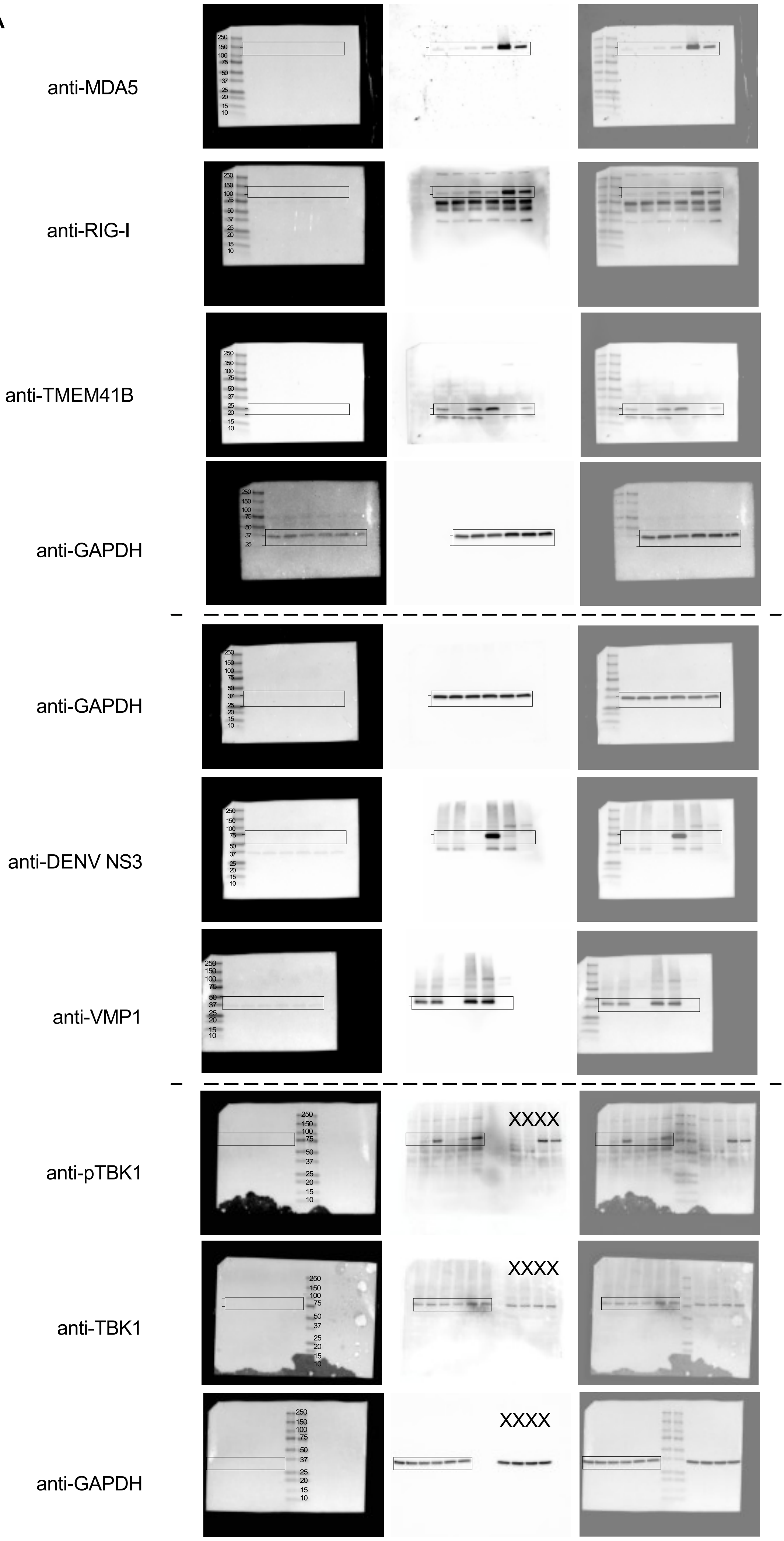
anti-MDA5
anti-RIG-I
anti-TMEM41B
anti-GAPDH
anti-GAPDH
anti-DENV NS3
anti-VMP1
anti-pTBK1
anti-TBK1
anti-GAPDH
XXXX
XXXX
XXXX

Figure 2B

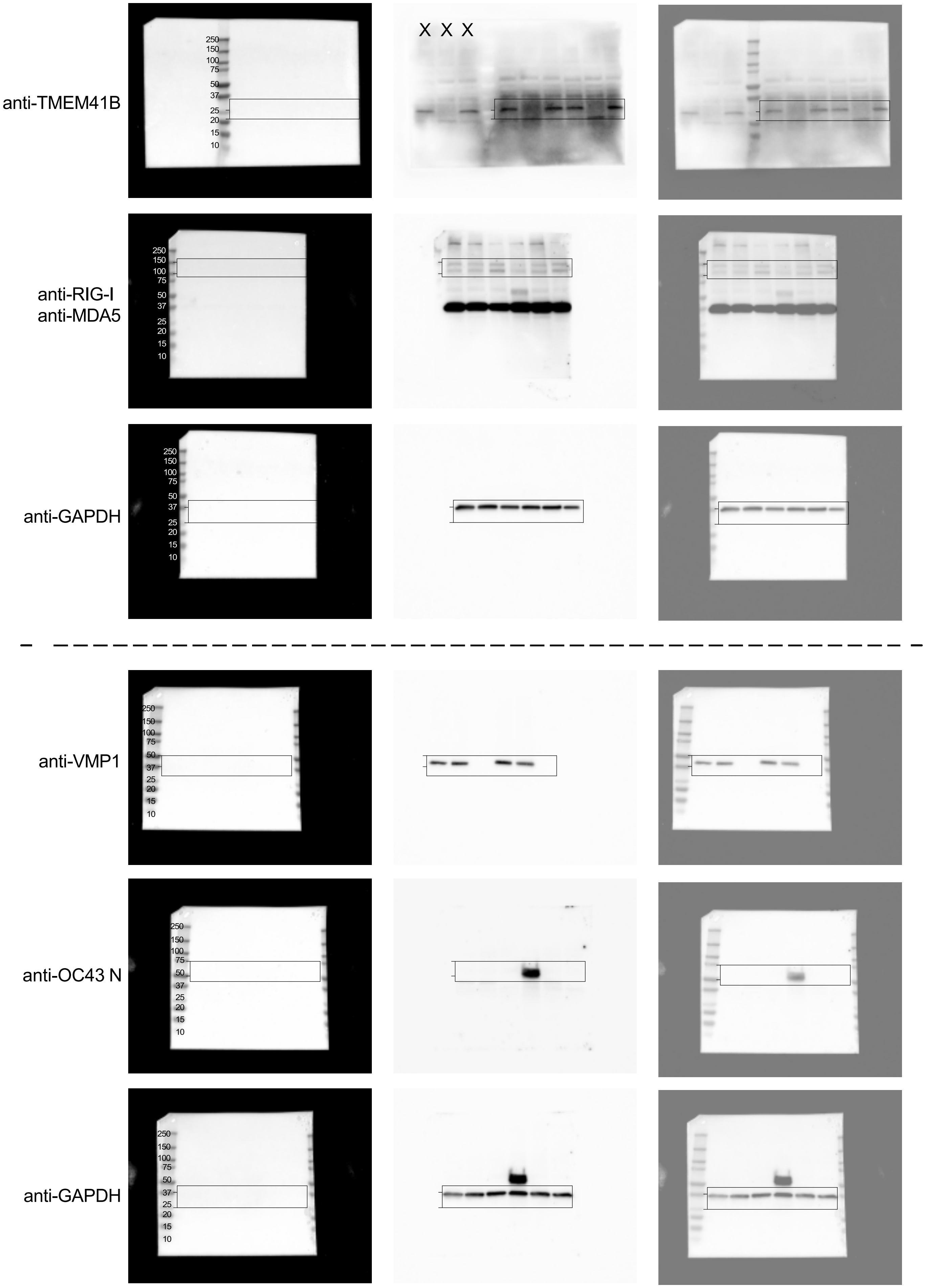

anti-TMEM41B
anti-RIG-I
anti-MDA5
anti-GAPDH
anti-VMP1
anti-OC43 N
anti-GAPDH
X X X
250
150
100
75
50
37
25
20
15
10

Figure 2F

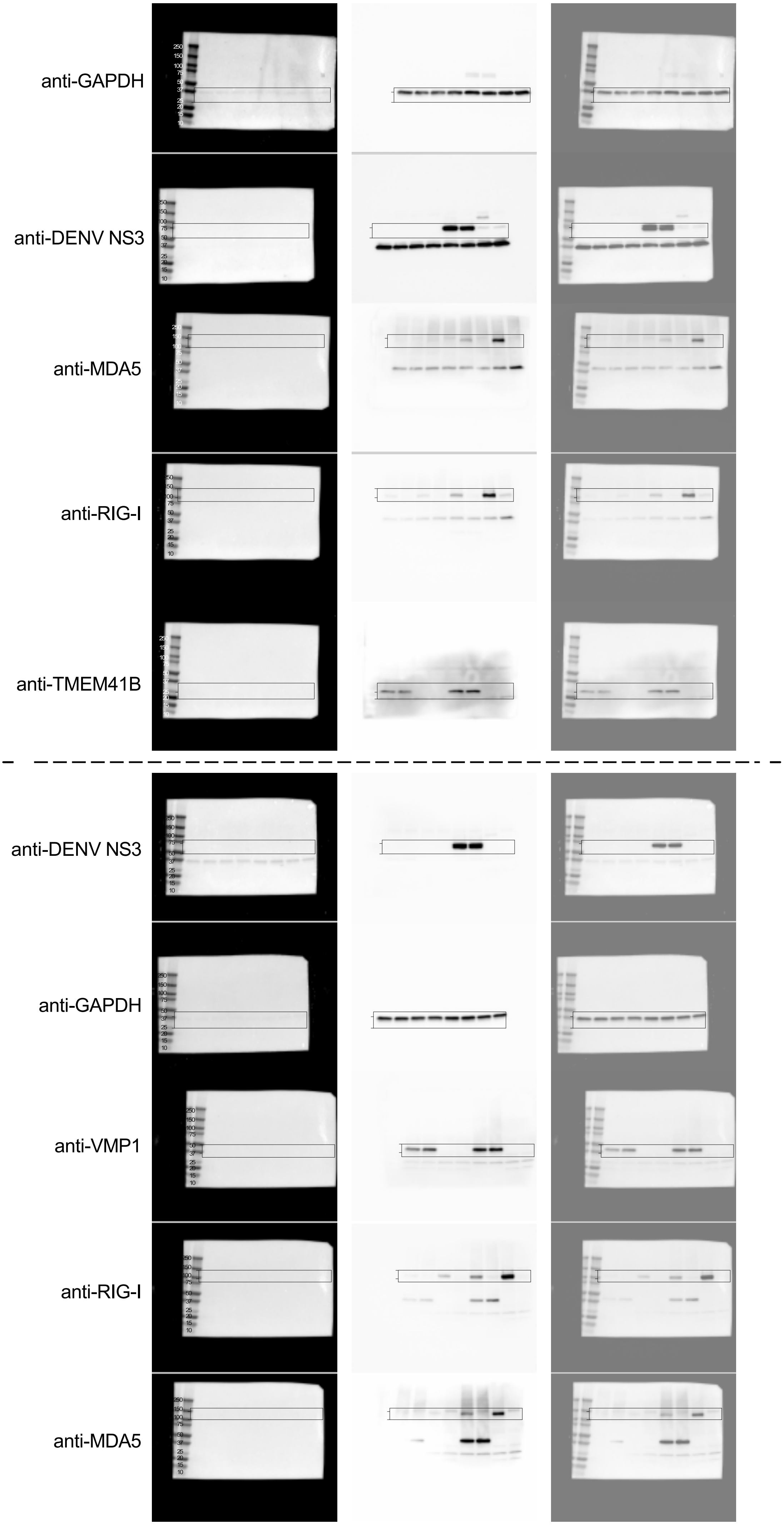
anti-GAPDH
anti-DENV NS3
anti-MDA5
anti-RIG-I
anti-TMEM41B
anti-DENV NS3
anti-GAPDH
anti-VMP1
anti-RIG-I
anti-MDA5

Figure 3D

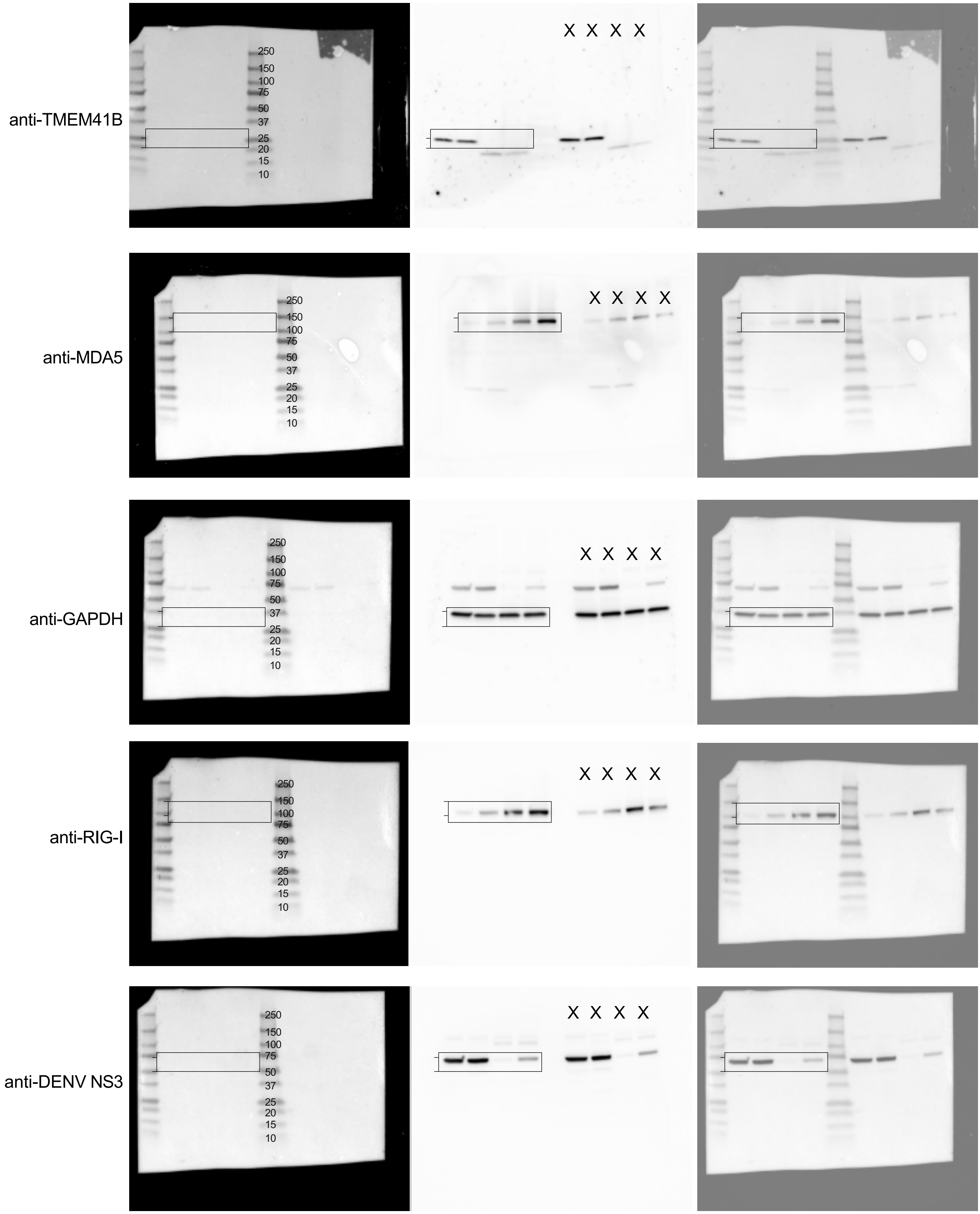
anti-TMEM41B
anti-MDA5
anti-GAPDH
anti-RIG-I
anti-DENV NS3
250
150
100
75
50
37
25
20
15
10
X X X X

S1 Fig A

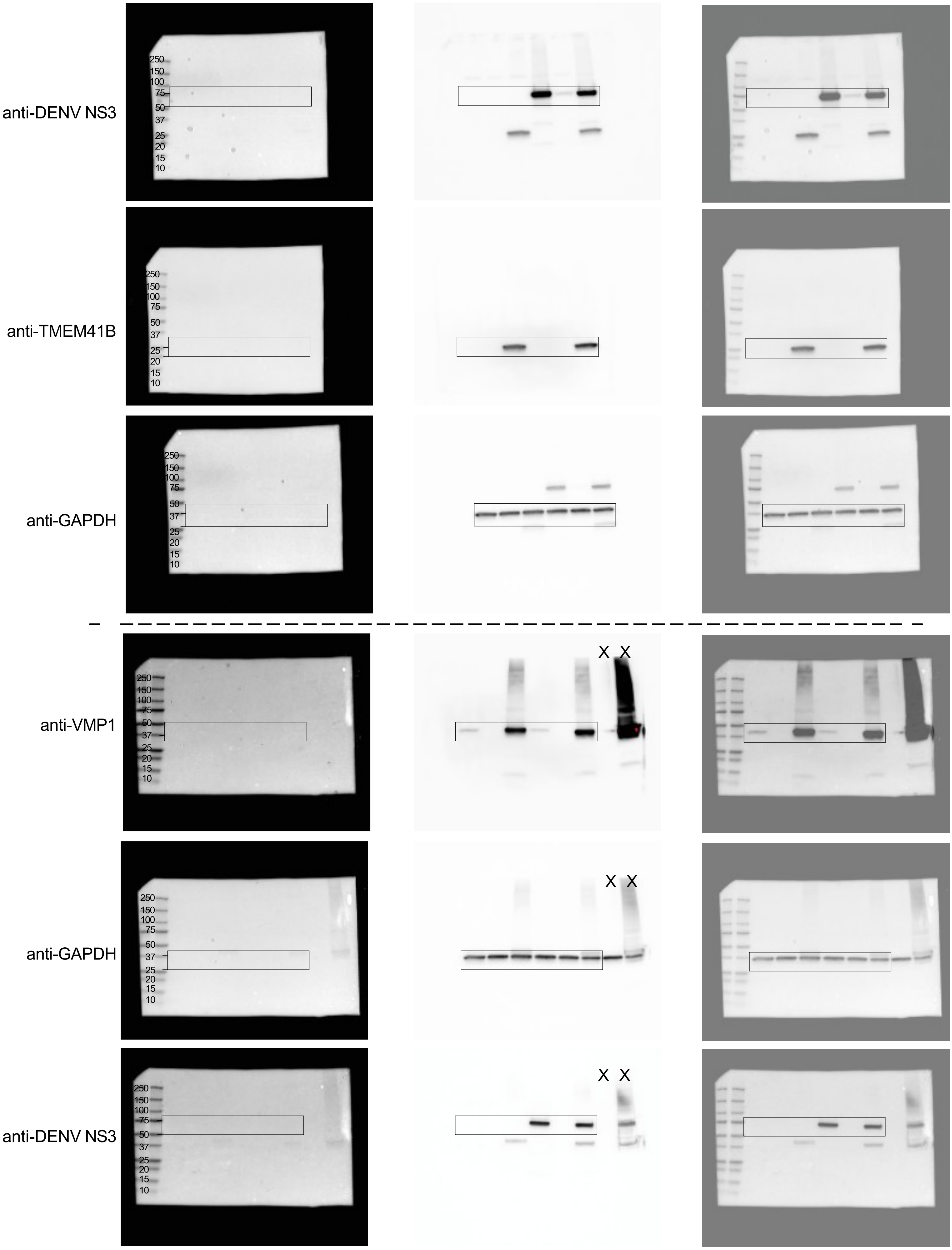

anti-DENV NS3
250
150
100
75
50
37
25
20
15
10
anti-TMEM41B
anti-GAPDH
anti-VMP1
X X
anti-GAPDH
anti-DENV NS3

S2 Fig

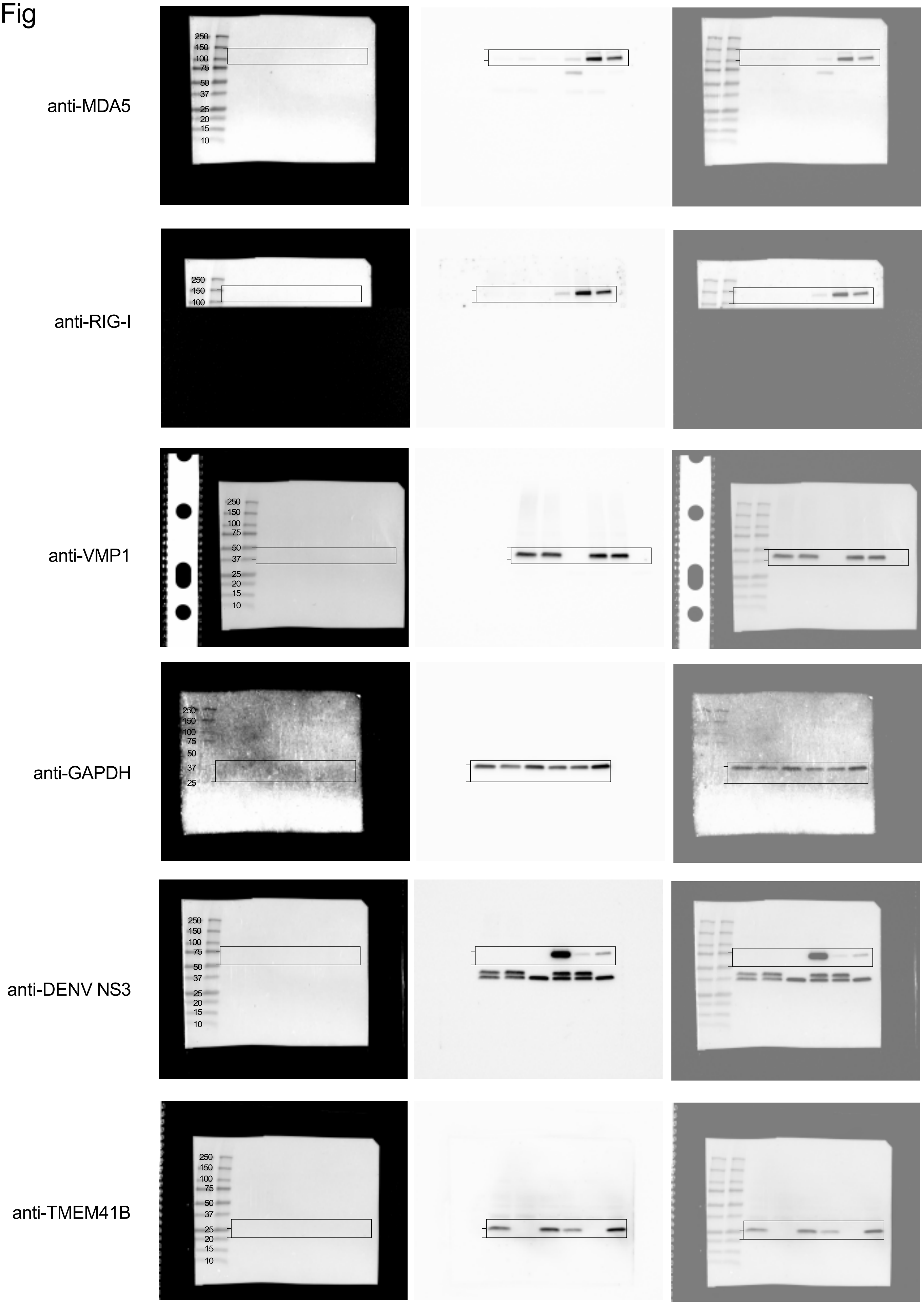
anti-MDA5
250
150
100
75
50
37
25
20
15
10
anti-RIG-I
250
150
100
anti-VMP1
250
150
100
75
50
37
25
20
15
10
anti-GAPDH
250
150
100
75
50
37
25
anti-DENV NS3
250
150
100
75
50
37
25
20
15
10
anti-TMEM41B
250
150
100
75
50
37
25
20
15
10
